# Investigating the Multitarget Mechanism of Traditional Chinese Medicine Prescription for Cancer-Related Pain by Using Network Pharmacology and Molecular Docking Approach

**DOI:** 10.1155/2020/7617261

**Published:** 2020-11-10

**Authors:** Jinyuan Chang, Lixing Liu, Yaohan Wang, Yutong Sui, Hao Li, Li Feng

**Affiliations:** ^1^National Cancer Center, National Clinical Research Center for Cancer, Cancer Hospital, Chinese Academy of Medical Sciences and Peking Union Medical College, Beijing 100021, China; ^2^Beijing University of Traditional Chinese Medicine, Beijing 100029, China

## Abstract

Gu-tong formula (GTF) has achieved good curative effects in the treatment of cancer-related pain. However, its potential mechanisms have not been explored. We used network pharmacology and molecular docking to investigate the molecular mechanism and the effective compounds of the prescription. Through the analysis and research in this paper, we obtained 74 effective compounds and 125 drug-disease intersection targets to construct a network, indicating that quercetin, kaempferol, and *β*-sitosterol were possibly the most important compounds in GTF. The key targets of GTF for cancer-related pain were Jun proto-oncogene (JUN), mitogen-activated protein kinase 1 (MAPK1), and RELA proto-oncogene (RELA). 2204 GO entries and 148 pathways were obtained by GO and KEGG enrichment, respectively, which proved that chemokine, MAPK, and transient receptor potential (TRP) channels can be regulated by GTF. The results of molecular docking showed that stigmasterol had strong binding activity with arginine vasopressin receptor 2 (AVPR2) and C-X3-C motif chemokine ligand 1 (CX3CL1) and cholesterol was more stable with p38 MAPK, prostaglandin-endoperoxide synthase 2 (PTGS2), and transient receptor potential vanilloid-1 (TRPV1). In conclusion, the therapeutic effect of GTF on cancer-related pain is based on the comprehensive pharmacological effect of multicomponent, multitarget, and multichannel pathways. This study provides a theoretical basis for further experimental research in the future.

## 1. Introduction

Cancer-related pain remains common and severe for many patients, especially in the advanced stage, while the prevalence is approximately more than 70% [1]. A large number of people suffer from mild to severe pain before they die, and only few patients with cancer pain are well managed because the pain is difficult to relieve. Cancer-related pain is an aggregation of many different cancer types, which is a mixed pain representing a homogenous pathological process, mainly including acute pain, inflammatory pain, nerve pain, and tumor-induced pain [2]. Currently, the principal strategy for cancer-related pain management has been based on an ordinal three-step analgesic ladder, but its clinical use is usually limited by the side effects of gastrointestinal bleeding, constipation, respiratory depression, and so on [3, 4].

In China, the application of Gu-tong Formula (GTF) in treatment of cancer-related pain has a history of decades, and it has been a patented prescription (Patent No.: 201410415620.1). GTF is prepared from a formula of nine Chinese medicines, including Radix Aconiti Lateralis Preparata (RALP), Cortex Cinnamomi (CC), Rhizoma Curculiginis (RC), Herba Asari (HA), Rhizoma Zingiberis (RZ), Radix Clematidis (CCO), Pseudobulbus Cremastrae seu Pleiones (PCSP), Scorpio (BMK), and Flos Caryophylli (FC). Preliminary clinical trials showed that GTF can observably reduce the frequency of breakthrough cancer pain and the requirements of medication [5]. However, the related mechanisms of its analgesic effect have not been entirely explored.

It is known that the Chinese herbal formula has a characteristic of multicomponent, multitarget, and multipathways; it means traditional experimental methods cannot detect its complex mechanisms absolutely. Network pharmacology, as a combination of pharmacology and pharmacodynamics, emphasizing the integration of disease, gene, target, and drug, has been widely used for exploring the overall effect of drugs on the treatment of diseases from a macroscopic and systematic point of view. Therefore, we adopt the network pharmacology approach to discover potential mechanisms of GTF in the treatment of cancer-related pain and use molecular docking for reverse verification.

## 2. Methods

### 2.1. Active Ingredients and Related Targets in GTF

#### 2.1.1. Pharmacokinetic Predictions

The active ingredients in GTF (except for BMK) were acquired from the Traditional Chinese Medicine Systems Pharmacology (TCMSP) database, a platform designed for herbs. Selecting oral bioavailability (OB) ≥30% and drug-likeness (DL) ≥0.18 as the screening criteria, the acquired active ingredients were searched for related protein targets from TCMSP and DrugBank databases [6, 7]. The active ingredients in BMK were obtained from the Traditional Chinese Medicine Information Database (TCMID) [8] and BATMAN-TCM [9]. Eventually, six effective compounds of BMK were collected, which were bufotoxin, chlorotoxin, katsutoxin, cholesterol, stearin, and 20-hexadecanoylingenol. Then, the chemical structure formula of the BMK active ingredients were downloaded from the PubChem database (https://pubchem.ncbi.nlm.nih.gov/), followed by importing to the PharmMapper database [10] and selecting the effective targets with high match (norm fit > 0.7).

#### 2.1.2. Potential Target Genes of Cancer-Related Pain

The data for the target genes of cancer-related pain were acquired from the DisGeNET database [11], GeneCards [12], and the Online Mendelian Inheritance in Man (OMIM) database [13]. All the data were standardized through the *UniProt* database [14].

1. DisGeNET database: search strategy—Set the disease name as “crushing pain, widespread chronic pain, postoperative pain, nerve pain, and intractable pain” in the *gene disease network* interface; 64 genes were collected (Table S1).
2. GeneCards: search strategy—Set the keyword as “cancer related pain” and the score >4 after logging into GeneCards; 1093 genes were collected (Table S2).
3. OMIM database: search strategy—Set the keyword as “cancer-related pain”; 22 genes were collected (Table S3).

#### 2.1.3. Drug-Disease-Target Network Construction

Intersection genes, which may treat the disease, were obtained by intersecting the drug targets and disease targets. We used Cytoscape v3.7.2 to construct the drug-compound-disease intersection gene network and then carried out topology analysis [15]. Finally, we calculated the degree value of each node in the network by *cytoHubba* plug-in to screen out key pharmacodynamic molecules [16].

#### 2.1.4. Protein-Protein Interaction (PPI) Data and Hub Gene Screening

We imported the intersection genes into the STRING database [17], and the species were set as *Homo sapiens*. The confidence score was set ≥0.9 to construct our PPI network [18], and the acquired network was imported into Cytoscape v3.7.2. The degree value of each node in the network was calculated using *cytoHubba* plug-in, and the top 10% was selected as the hub gene. Then, the biological process of GO was analyzed for the hub gene.

### 2.2. Enrichment Analysis

#### 2.2.1. Gene Ontology (GO) and Kyoto Encyclopedia of Genes and Genomes (KEGG) Pathway Enrichment Analysis

ClusterProfiler package was used to perform GO and KEGG enrichment analysis of the intersection gene with *p* < 0.05 [19].

#### 2.2.2. Molecular Docking Verification

AutoDock was used to perform the receptor-ligand docking simulation calculation of key pharmacodynamic molecules and screened core targets. The protein structure, downloaded from the PDB database, was imported into POCASA v1.1 [20]. Meanwhile, the position of the active site was investigated in the PubMed database to verify the predicted position by POCASA v1.1. The docking operation used the Lamarckian genetic algorithm, and the rigid receptor-flexible ligand docking pattern was used in the docking process. The number of runs was 50, and the maximum energy evaluation was 2,500,000 [21]. The ligand corresponding to the target protein was used as a positive control. After all the docking simulations were completed, heat maps were made according to the strongest affinity of key pharmacodynamic molecules and core targets.

## 3. Results and Discussion

**Figure 1:**
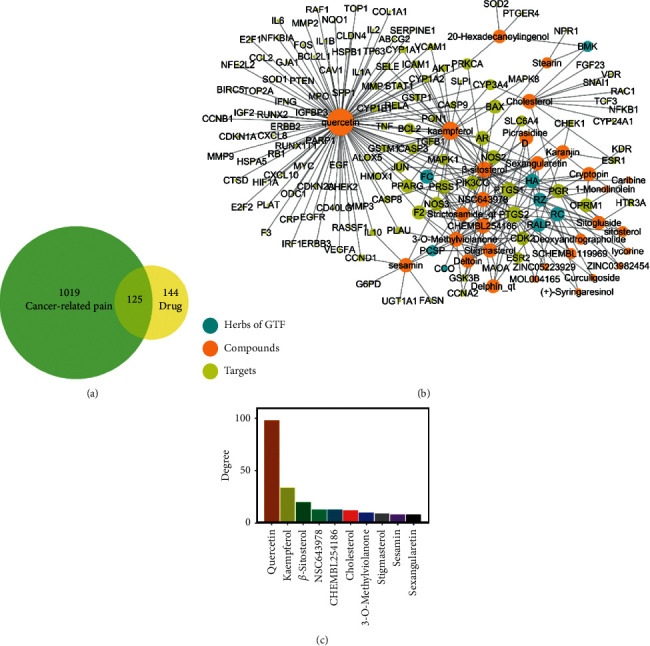
(a) Venn map of disease-related targets and drug targets. (b) Gu-tong formula (GTF) drug-disease-target network. Radix Aconiti Lateralis Preparata (RALP), Cortex Cinnamomi (CC), Rhizoma Curculiginis (RC), Herba Asari (HA), Rhizoma Zingiberis (RZ), Radix Clematidis (CCO), Pseudobulbus Cremastrae seu Pleiones (PCSP), Scorpio (BMK), and Flos Caryophylli (FC). (c) The top ten key pharmacodynamic molecules with the degree value.

In this study, we obtained 74 active compounds (Table S4) and 269 potential targets of GTF after deleting duplicates. The active compound-related targets in TCMSP and PharmMapper databases are listed in Tables S5 and S6, respectively. 1144 potential target genes of cancer-related pain were collected, and we obtained 125 drug-disease intersection targets (Figure 1(a)).

### 3.1. GTF Drug-Disease-Target Network

The intersection targets were imported into Cytoscape v3.7.2 to construct a drug-disease-target network (Figure 1(b)), including 163 nodes and 296 edges, with a network heterogeneity of 2.336 and a network centralization of 0.590. It indicated that some nodes in the network were more concentrated than others. The network showed that compounds with most high connectivity were quercetin (degree = 98), kaempferol (degree = 34), and *β*-sitosterol (degree = 20), which were sorted by the degree value, suggesting that these three compounds were possibly the most important compounds in GTF (Figure 1(c)). Quercetin and kaempferol are flavonoids, and *β*-sitosterol is a phytosterol. All of them can regulate the way of cell apoptosis, proliferation, and phosphoinositide 3-kinase (PI3K)/protein kinase B (AKT) pathways to achieve the purpose of antitumor [22–24]. Moreover, they also have a strong inhibitory effect on inflammation through the inhibition of lipoxygenase and cyclooxygenase pathways. Among them, kaempferol has been used as a chemosensitizer in clinical research and has the potential of reducing toxicity and enhancing efficacy [25].

We preliminary analyzed the targets of the compounds in the network. NPR1 with a 0.672 disease-specific index (DSI) was targeted by stearin (extracted from BMK) and was important in crushing pain. ORPM1, which was targeted by *β*-sitosterol (extracted from FC, RZ, PCSP, CCO, and RC), caribine, cryptopine (extracted from HA), and lycorine (extracted from RC), played an important role in nerve pain, and its DSI was 0.479. The DSI of CXCL8 targeted by quercetin (extracted from FC) in intractable pain was 0.342. These three types of pain were crucial in cancer-related pain.

### 3.2. PPI Network

**Figure 2:**
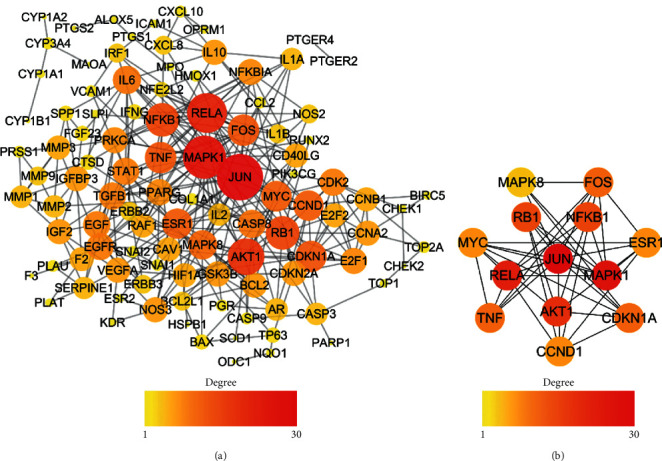
(a) PPI network of the intersection targets. (b) The hub genes in the PPI network.

The intersection targets were imported into the STRING 11.0 database, and then, 104 nodes and 324 edges can be obtained, with an average degree of 5.18 for each node and an average neighbor of 6.231 for each edge (Figure 2(a)). As shown in Figure 2(b), the hub genes in the network screened by *cytoHubba* plug-in were JUN (degree = 28), MAPK1 (degree = 25), RELA (degree = 22), etc. Among them, RELA, RB1, NFKB1, TNF, and ESR1 had the function of regulating inflammatory response (GO: 0050727) and cellular response to reactive oxygen species (GO: 0034614). JUN, AKT1, FOS, and MAPK8 played the role of response to mechanical stimulus (GO: 0009612).

TNF mainly encoded a multifunctional proinflammatory cytokine, combination with the transient receptor potential vanilloid-1 (TRPV1), playing an important role in inflammatory pain and nerve pain [26, 27]. Downregulating TNF expression can effectively inhibit the occurrence of inflammatory pain and nerve pain [28]. Moreover, MAPK8, JUN, AKT1, and RELA can regulate cell proliferation and cell cycle, which was crucial in tumorigenesis. GTF can regulate the expression of these genes to affect the development of tumors.

### 3.3. GO Enrichment

**Figure 3:**
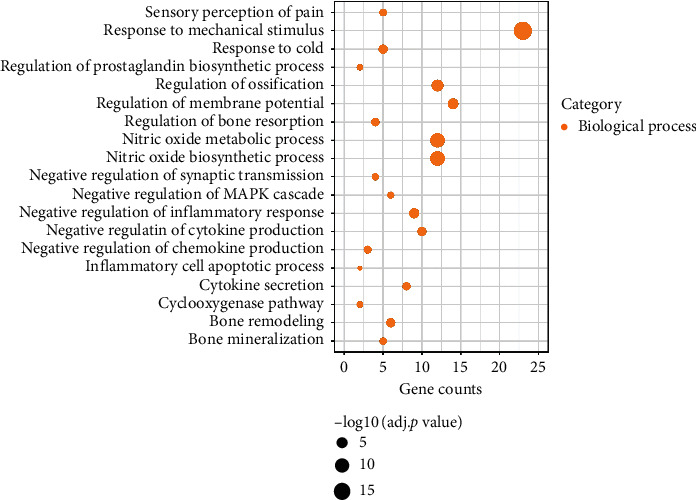
Gene Ontology (GO) enrichment result of GTF in treatment of cancer-related pain. The size of the bubble represents the different adjusted *p* value.

To further explore the multiple mechanisms of GTF, GO enrichment analysis (take biological process for example) was performed (Figure 3), and 2204 GO entries were enriched.

We found that GTF was involved in regulating the production of neuronal action potential via the regulation of membrane potential (GO: 0042391) and synaptic transmission (GO: 0050805). The activation of neurons and the conduction of electrical signals were the fundamental causes of nerve sensitization and pain [29]. GTF inhibited the conduction of pain by means of controlling the transmission of neurotransmitters to maintain the stability of the postsynaptic membrane.

What is more, GTF inhibited the secretion of inflammatory factors in the inflammatory response by means of regulating the prostaglandin (PG) biosynthetic process (GO: 0031392), cyclooxygenase pathway (GO: 0019371), inflammatory response (GO: 0050728), and cytokine production (GO: 0001818). Among them, PG induced inflammation and hyperalgesia, leading to the occurrence and aggravation of pain. In the first stage of three-step pain relief, it achieved the purpose of pain relief by inhibiting the target and cyclooxygenase pathway [2]. GTF also played a role in this pathway and the target and inhibited the occurrence of inflammatory pain. Moreover, it relieved the pain caused by tumor compression and sensitivity to cold stimulation after chemotherapy via the response to mechanical stimulus (GO: 0009612) and cold (GO: 0009409). Activation of the MAPK pathway was crucial in the development of pain [30], and GTF-negative regulated the MAPK cascade (GO: 0043409) to restrain the occurrence of pain.

Cancer-related pain caused by bone metastasis and bone destruction is usually difficult to relieve [31]. It was believed that bone resorption and bone formation were the key factors of bone destruction caused by tumor [32]. Interestingly, we found that GTF protected bones through regulation of ossification (GO: 0030278), bone resorption (GO: 0045124), and remodeling (GO: 0046849). Detailed GO enrichment information is shown in Table 1. The multitarget and multifunctional characteristics of GTF played a certain role in alleviation of cancer-related pain by regulating nerves, reducing inflammation and mechanical stimulation, and protecting bone.

### 3.4. KEGG Enrichment

We obtained 148 pathways in total, which belonged to several categories, including tumor, inflammation, infection, and other pathways. After analyzing the results of KEGG enrichment, we found that GTF mainly focused on the regulation of proinflammatory cytokines, damage associated molecular pattern (DAMP), neural sensitization, and pain-related protein kinase (Table 2).

GTF can regulate the synthesis and secretion of inflammatory factors by regulating IL-17 (hsa04657), TNF (hsa04668), and chemokine signaling pathways (hsa04062). IL-17, secreted by CD4^+^ T cells, can induce epithelial cells and endothelial cells to synthesize IL-6, PG, and other cytokines, and combination with the TNF signaling pathway to promote inflammation [33]. Inhibition of IL-17 can effectively relieve pain and inhibit peripheral nerve sensitization [34]. C-X3-C motif chemokine ligand 1 (CX3CL1) is a kind of chemokine. It was found that CX3CL1 and its receptor CX3CR1 were very important in the development of neuropathic pain [35]. Soluble CX3CL1 bound to CX3CR1 on the surface of microglia, which lead to the increase of intracellular calcium concentration and the occurrence of neuropathic pain [36, 37]. In the study of network pharmacology of GTF, it was shown that the application of GTF can affect those pathways and inhibit the occurrence of pain. At the same time, we found that GTF can regulate the response of the body to DAMP by regulating Toll-like receptor (hsa04620), NOD-like receptor (hsa04621), and RIG-I-like receptor signaling pathways (hsa04622). It had been proven that Toll-like receptors were highly expressed in microglia of mice with neuropathy. When the Toll-like receptor signaling pathway was inhibited, it can effectively inhibit the occurrence of pain [38].

In the aspect of neurotransmitter transmission and central sensitization, GTF can regulate the release of neurotransmitters and maintain the stability of neuron membrane potential by influencing serotonergic (hsa04726) and dopaminergic synapse (hsa04728). Transient receptor potential (TRP) was widely distributed in nociceptive neurons and was related to the persistence of pain [39]. GTF regulated this pathway (hsa04750) to affect pain duration.

p38 MAPK was widely distributed in the spinal dorsal horn and can be activated by various external stimuli such as trauma stimulation and inflammatory factors [40] and participated in the occurrence of pain through phosphorylated voltage-gated sodium channels. The JAK-STAT pathway was activated by interleukin-6 (IL-6) involving in the occurrence of inflammation and pain [41]. GTF can regulate the release of PG and excitatory amino acids by regulating these two pathways, to reduce cancer pain.

### 3.5. Molecular Docking

POCASA v1.1 was used to predict the most likely docking pocket of protein, which was sorted according to the volume of the pocket (Figure S1, docking pocket from large to small: a–e). In addition, compared with the active sites reported in the PubMed database, the docking sites of protein targets were obtained. The detailed information for core protein is listed in Table S7.

**Figure 4:**
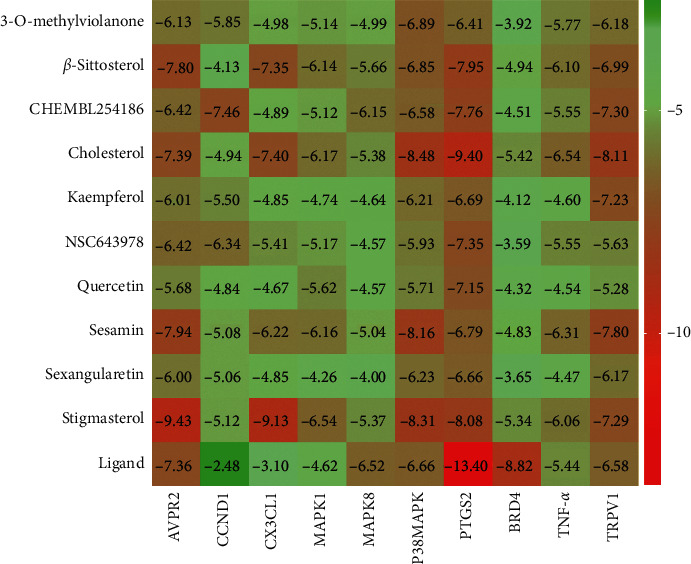
The binding energy of 10 key drug molecules and 10 target proteins.

Figure 4 shows the mapping of the strongest affinity of 10 key drug molecules and 10 core target proteins. We found that the binding energy between the molecule and the target protein was approximately between −3.59 and −9.43 kcal·mol^−1^. It can be seen from Figure 4 that AVPR2, CX3CL1, p38 MAPK, prostaglandin-endoperoxide synthase 2 (PTGS2), and TRPV1 have stronger docking energy. It means that the compounds in GTF bind well to the above target proteins.

Arginine vasopressin (AVP), an important analgesic substance, can be synthesized and secreted by the paraventricular nucleus of the hypothalamus [42]. After external stimulation, the expression of AVP increased and transported to the midbrain aqueduct gray matter, nucleus raphe magnus, caudate nucleus, and other related nuclei, resulting in the secretion of endogenous opioid peptide, 5-hydroxytryptamine and acetylcholine, and activating vasopressin receptors in central and peripheral tissues, which played a crucial role in nociception. Studies had shown that high dose of AVP can increase the action potential of C-type nociceptive fibers and produce analgesic effect [43]. The receptors of AVP, including V1a, V1b, and V2, belong to G protein-coupled receptor [44]. The compounds contained in GTF can stably bind to G protein-coupled receptor, producing analgesic effect.

CX3CL1 and its receptor CX3CR1 were both expressed in the nervous system [35] and played a role in promoting the occurrence of pain, which had been confirmed in many experiments. The researchers believed that the painful behavior caused by CX3CL1 was achieved by exciting CX3CR1 on microglia and activating the p38 MAPK signaling pathway [45]. Interestingly, our molecular docking results showed that GTF had better binding effect with CX3CL1 and p38 MAPK. In the pain model of bone tumor in rats made by injecting Walker 256 breast cancer cells into the tibial marrow cavity, intrathecal injection of CX3CR1 neutralizing antibody can reduce the development of pain and hyperalgesia, and blocking CX3CR1 can inhibit the activation of spinal microglia and the phosphorylation level of p38 MAPK [46]. It indicated the role of CX3CL1/CX3CR1/p38 MAPK pathway in the formation and development of cancer pain. This further showed that GTF can affect the occurrence and development of pain by regulating the above pathways.

PTGS2, also known as cyclooxygenase-2 (COX-2), was the second isozyme of cyclooxygenase. COX was an important rate-limiting enzyme for PG. COX-2 can be induced by a variety of inflammatory mediators and cytokines and participated in tissue inflammation and cell differentiation and proliferation. Upregulation of COX-2 was also related to antiapoptosis and tumor angiogenesis [47]. The results of molecular docking showed that the pharmacodynamic molecules selected in GTF had stronger binding energy to COX-2, which meant that the active ingredients in GTF can regulate the development of inflammatory pain and tumors through COX-2.

TRPV1 was a heat-activated cation channel protein, which was expressed on primary afferent neurons and upregulated after inflammation and nerve injury and was closely related to inflammation and acute and chronic pain [48]. Inhibition of TRPV1 activity was one of the pain treatment methods. In the preclinical study, it has the potential to be a receptor of nonopioid analgesics [49]. The results of molecular docking showed that the compounds contained in GTF can better combine with TRPV1 and played an analgesic role.

**Figure 5:**
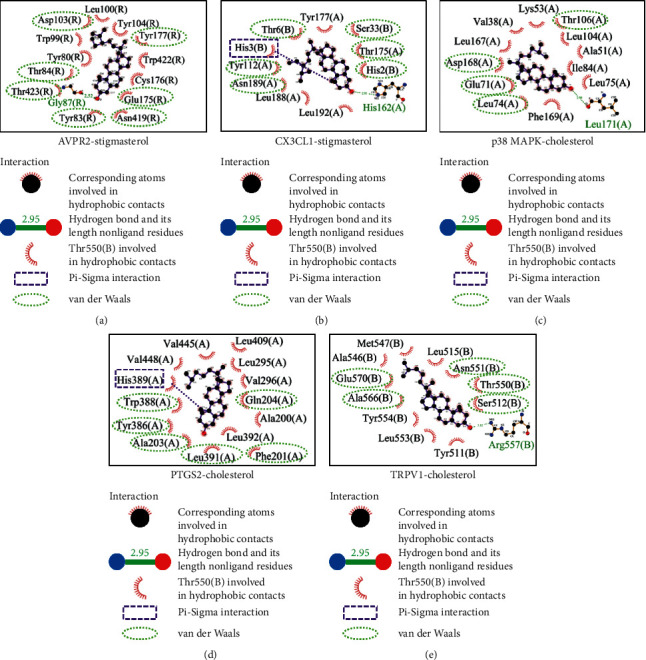
Binding mode of protein and compounds.

According to the docking energy results, we selected the 2 compounds with the strongest protein docking results from the 5 proteins, respectively. Stigmasterol had the strongest affinity with AVPR2, CX3CL1, and cholesterol with PTGS2, p38 MAPK, and TRPV1. The binding patterns of compounds and proteins were plotted, and then the interaction between compounds and binding sites and surrounding amino acid residues was observed (Figure 5). Cholesterol and stigmasterol both occupied the docking pocket of the protein and bound stably. The analysis of the docking effect of compounds and receptor proteins is shown in Table 3.

All the five protein targets are able to form hydrophobic interaction and van der Waals force with compounds and stable binding. Stigmasterol (with AVPR2 and CX3CL1) and cholesterol (with p38 MAPK and TRPV1) can form hydrogen bonds on Gly87, His162, Leu171, and Arg557 residues, respectively, which made the binding more robust. CX3CL1 (with stigmasterol) and PTGS2 (with cholesterol) can form Pi-Sigma interaction on His3 and His389 residues, respectively, which increased the binding stability.

Stigmasterol is a common phytosterol. Modern pharmacological studies showed that it can effectively relieve acute and chronic pain and had neuroprotective effect on ischemic/reperfusion injury and good anti-inflammatory effect [50, 51]. In the process of molecular docking, the stigmasterol also had strong binding activity with AVPR2 and CX3CL1. It also suggested that stigmasterol in GTF was also an effective component in relieving cancer pain.

Cholesterol, widely consisted in vivo, a precursor of neurosteroid biosynthesis, is an endogenous produced molecule that inhibits TRPV1 activity and plays an important role in the regulation of nervous system injury and disorder [52]. Little is known about its role in neuropathic pain. Recent studies have shown that in inflammatory animal models, transcutaneous cholesterol delivery can alleviate allergic reactions and cholesterol homeostasis can help regulate inflammation and pain [53]. Increasing cholesterol content can inhibit the expression of PTGS2 and the secretion of PG [54], while decreasing cholesterol content can enhance p38 MAPK and inflammatory activity [55]. In molecular docking research, we found that cholesterol contained in GTF can stably target PTGS2, p38 MAPK, and TRPV1, which may play an analgesic role through MAPK and chemokine pathways.

## 4. Conclusion

To sum up, we revealed the potential pharmacological mechanism of GTF in the treatment of cancer pain from a systematic perspective, which may involve the secretion of inflammatory cytokines, membrane potential, bone protection, and other biological processes by regulating chemokine, MAPK, and TRP channels. The cholesterol and stigmasterol in GTF can be the key pharmacodynamic molecules for analgesia in molecular docking screening. This study provides clues for understanding the synergistic effect of GTF in relieving cancer pain. However, considering that this study is mainly based on data analysis and molecular docking, further experiments are necessary to verify the results.

## Figures and Tables

**Table 1:** Gene Ontology (GO) enrichment result.

ID	Description	*p* value	Count
GO:0009612	Response to mechanical stimulus	2.60 × 10^−19^	23
GO:0006809	Nitric oxide biosynthetic process	5.86 × 10^−12^	12
GO:0046209	Nitric oxide metabolic process	1.24 × 10^−11^	12
GO:0030278	Regulation of ossification	1.55 × 10^−7^	12
GO:0042391	Regulation of membrane potential	3.96 × 10^−6^	14
GO:0050728	Negative regulation of inflammatory response	1.41 × 10^−5^	9
GO:0009409	Response to cold	1.09 × 10^−4^	5
GO:0001818	Negative regulation of cytokine production	1.52 × 10^−4^	10
GO:0046849	Bone remodeling	1.55 × 10^−4^	6
GO:0045124	Regulation of bone resorption	5.98 × 10^−4^	4
GO:0050663	Cytokine secretion	8.37 × 10^−4^	8
GO:0032682	Negative regulation of chemokine production	1.27 × 10^−3^	3
GO:0019233	Sensory perception of pain	2.17 × 10^−3^	5
GO:0030282	Bone mineralization	3.11 × 10^−3^	5
GO:0050805	Negative regulation of synaptic transmission	4.09 × 10^−3^	4
GO:0043409	Negative regulation of MAPK cascade	4.39 × 10^−3^	6
GO:0019371	Cyclooxygenase pathway	6.38 × 10^−3^	2
GO:0031392	Regulation of prostaglandin biosynthetic process	7.40 × 10^−3^	2
GO:0006925	Inflammatory cell apoptotic process	2.02 × 10^−2^	2

**Table 2:** Kyoto Encyclopedia of Genes and Genomes (KEGG) enrichment result.

ID	KEGG pathway	*p* value	Count
hsa04657	IL-17 signaling pathway	4.64 × 10^−18^	21
hsa04668	TNF signaling pathway	9.66 × 10^−18^	22
hsa04620	Toll-like receptor signaling pathway	4.63 × 10^−12^	16
hsa04010	MAPK signaling pathway	3.76 × 10^−11^	24
hsa04621	NOD-like receptor signaling pathway	2.40 × 10^−7^	15
hsa04630	JAK-STAT signaling pathway	3.94 × 10^−7^	14
hsa04726	Serotonergic synapse	2.15 × 10^−5^	10
hsa04622	RIG-I-like receptor signaling pathway	2.35 × 10^−5^	8
hsa04062	Chemokine signaling pathway	6.33 × 10^−5^	12
hsa04728	Dopaminergic synapse	2.40 × 10^−2^	6
hsa04750	Inflammatory mediator regulation of TRP channels	2.84 × 10^−2^	5

**Table 3:** Docking effect analysis of compounds and targets.

Compound	Target	Binding energy (kcal/mol)	Hydrogen bonding interaction	Hydrophobic interaction
Stigmasterol	AVPR2	−9.43	Cly87	Tyr80, Tyr83, Tyr84, Trp99, Leu100, Asp103, Tyr104, Glu175, Cys176, Tyr177, Asn419, Trp422, Thr423
	CX3CL1	−9.13	His162	Chain (A): Tyr112, Thr175, Tyr177, Leu188, Asn189, Leu192; China (B): His2, His3, Thr6, Ser33
Cholesterol	p38 MAPK	−8.48	Leu171	Val38, Ala51, Lys53, Glu71, Leu74, Leu75, Ile84, Leu104, Thr106, Leu167, Asp168, Phe169
	PTGS2	−9.4	Not formed	Ala200, Phe201, Ala203, Gln204, Leu295, Val296, Tyr386, Tyr388, His389, Leu391, Leu392, Leu409, Val445, Val448
	TRPV1	−8.11	Arg557	Tyr511, Ser512, Leu515, Ala546, Met547, Thr550, Asn551, Leu553, Tyr554, Ala566, Glu570

## Data Availability

The data used to support the findings of this study are included within the article and the supplementary information files.

## Abbreviations

GTF: Gu-tong formula
JUN: Jun proto-oncogene
MAPK: Mitogen-activated protein kinase
RELA: RELA proto-oncogene
AVPR2: Arginine vasopressin receptor 2
CX3CL1: C-X3-C motif chemokine ligand 1
PTGS2: Prostaglandin-endoperoxide synthase 2
TRPV1: Transient receptor potential vanilloid-1
RALP: Radix Aconiti Lateralis Preparata
CC: Cortex Cinnamomi
RC: Rhizoma Curculiginis
HA: Herba Asari
RZ: Rhizoma Zingiberis
CCO: Radix Clematidis
PCSP: Pseudobulbus Cremastrae seu Pleiones
BMK: Scorpio
FC: Flos Caryophylli
TCMSP: Traditional Chinese Medicine Systems Pharmacology
OB: Bioavailability
DL: Drug-likeness
TCMID: Traditional Chinese Medicine Information Database
BATMAN-TCM: A Bioinformatics Analysis Tool for Molecular mechANism of Traditional Chinese Medicine
OMIM: Online Mendelian Inheritance in Man
PPI: Protein-protein interaction
GO: Gene Ontology
KEGG: Kyoto Encyclopedia of Genes and Genomes
PI3K/AKT: Phosphoinositide 3-kinase/protein kinase B
PG: Prostaglandin
DSI: Disease-specific index
NPR1: Natriuretic peptide receptor 1
OPRM1: Opioid receptor mu 1
CXCL8: Interleukin-8
RB1: Retinoblastoma-associated protein
CDKN1A: Cyclin-dependent kinase inhibitor 1
FOS: Proto-oncogene c-Fos
TNF: Tumor necrosis factor
ESR1: Estrogen receptor
MYC: Myc proto-oncogene protein
DAMP: Damage associated molecular pattern
IL-6: Interleukin-6
JAK-STAT: Janus kinase-signal transducer and activator of transcription
COX-2: Cyclooxygenase-2.

## Supplementary Materials

Supplementary Materials Figure S1. The docking pocket of target proteins predicted by POCASA v1.1, and the order of pocket volume is from large to small: a–e. Table S1. The targets of cancer-related pain in DisGeNET. Table S2. The targets of cancer-related pain in GeneCards. Table S3. The targets of cancer-related pain in OMIM. Table S4. All the GTF compounds. Table S5. Potential targets of GTF compounds downloaded from TCMSP database. Table S6. Potential targets of BMK active compounds downloaded from PharmMapper database. Table S7. The PDB information for core protein.
