# Supplementary material for: Investigating the Multitarget Mechanism of Traditional Chinese Medicine Prescription for Cancer-Related Pain by Using Network Pharmacology and Molecular Docking Approach
Source: Evid Based Complement Alternat Med. 2020 Nov 10;2020:7617261. doi: 10.1155/2020/7617261 (PMC7673937; doi:10.1155/2020/7617261)
Supplement: Supplementary Materials — Figure S1. The docking pocket of target proteins predicted by POCASA v1.1, and the order of pocket volume is from large to small: a–e. Table S1. The targets of cancer-related pain in DisGeNET. Table S2. The targets of cancer-related pain in GeneCards. Table S3. The targets of cancer-related pain in OMIM. Table S4. All the GTF compounds. Table S5. Potential targets of GTF compounds downloaded from TCMSP database. Table S6. Potential targets of BMK active compounds downloaded from PharmMapper database. Table S7. The PDB information for core protein. [file 7617261.f1.docx]

**Supporting Information**

Investigating the multi-target mechanism of traditional Chinese medicine prescription for cancer related pain: by using network pharmacology and molecular docking approach

*Jinyuan Chang^1^ Lixing Liu^1^ Yaohan Wang^1^ Yutong Sui^1^ Hao Li^2^ and Li Feng^1^ **

*^1^National Cancer Center/National Clinical Research Center for Cancer/Cancer Hospital, Chinese Academy of Medical Sciences and Peking Union Medical College, Beijing (100021), China*

*^2^ Beijing university of Traditional Chinese Medicine, Beijing (100029), China*

** Correspondence: fengli663@126.com;*

**The Potential Target Genes of cancer related pain**

The Potential Target Genes of cancer related pain were acquired from DisGeNET database (Table S1), Genecards (Table S2), and the Online Mendelian Inheritance in Man database (OMIM) (Table S3).

In the Table S1, the search strategy was setting the disease name as “crushing pain, widespread chronic pain, postoperative pain, nerve pain and intractable pain” in gene disease network interface.

In the Table S2, the search strategy was setting the keyword as “cancer related pain” and the score >4 after logging in to Genecards.

In the Table S3, the search strategy was setting the keyword as “cancer related pain”.

**The Pharmacokinetic Predictions of Gu-tong formula (GTF).**

The Pharmacokinetic Predictions of GTF (except for Scorpio) were acquired from the Traditional Chinese Medicine Systems Pharmacology Database (TCMSP), and Selecting the oral bio-availability (OB) ≥30% and drug-likeness (DL) ≥0.18 as the screening criteria. The active ingredients in Scorpio were obtained from Traditional Chinese Medicine Information Database (TCMID) and BATMAN-TCM. After the deleting of duplicates, we obtained 74 effective compounds in total (Table S4).

**The potential targets of GTF compounds**

The potential targets of active ingredients in GTF (except for Scorpio) were downloaded from Traditional Chinese Medicine Systems Pharmacology Database (TCMSP) (Table S5), and the Scorpio related targets were harvested from Pharmmapper database with high match (Norm Fit >0.7) (Table S6).

**The detailed information for core protein**

Investigating the position of the active site in the Pubmed database and referring to the predicted position of the POCASA v1.1. The PDB information for core protein is listed in Table S7. The protein structure is downloaded from PDB database (https://www.rcsb.org/).

**The docking pocket of target proteins predicted by POCASA v1.1.**

Importing the protein structure downloaded from the PDB database into the POCASA v1.1, predicting the protein docking pockets, and sorted by the volume of the pocket (Figure S1, docking pockets from large to small: a-e).

**Table S1.** The targets of cancer related pain in DisGeNet

| Disease Name | Gene ID | Symbol |
| --- | --- | --- |
| Pain, Crushing/ Nerve Pain | 6863 | TAC1 |
| Pain, Crushing | 3827 | KNG1 |
| Pain, Crushing | 1440 | CSF3 |
| Pain, Crushing | 3269 | HRH1 |
| Pain, Crushing | 3558 | IL2 |
| Pain, Crushing | 3350 | HTR1A |
| Pain, Crushing | 4878 | NPPA |
| Pain, Crushing | 3557 | IL1RN |
| Pain, Crushing | 5179 | PENK |
| Pain, Crushing | 1813 | DRD2 |
| Pain, Crushing | 3060 | HCRT |
| Pain, Crushing | 23236 | PLCB1 |
| Pain, Crushing | 1392 | CRH |
| Pain, Crushing | 10887 | PROKR1 |
| Pain, Crushing | 7442 | TRPV1 |
| Pain, Crushing | 133 | ADM |
| Pain, Crushing | 207 | AKT1 |
| Pain, Crushing/ Nerve Pain | 781 | CACNA2D1 |
| Pain, Crushing | 5020 | OXT |
| Pain, Crushing | 9177 | HTR3B |
| Pain, Crushing | 3363 | HTR7 |
| Pain, Crushing | 5173 | PDYN |
| Pain, Crushing | 2902 | GRIN1 |
| Pain, Crushing/ Nerve Pain | 7124 | TNF |
| Pain, Crushing | 2643 | GCH1 |
| Pain, Crushing | 1268 | CNR1 |
| Pain, Crushing | 5368 | PNOC |
| Pain, Crushing | 6548 | SLC9A1 |
| Pain, Crushing | 1906 | EDN1 |
| Pain, Crushing | 358 | AQP1 |
| Pain, Crushing | 5443 | POMC |
| Pain, Crushing | 624 | BDKRB2 |
| Pain, Crushing | 623 | BDKRB1 |
| Pain, Crushing | 27185 | DISC1 |
| Pain, Crushing | 3084 | NRG1 |
| Pain, Crushing | 1312 | COMT |
| Pain, Crushing | 8989 | TRPA1 |
| Pain, Crushing | 4923 | NTSR1 |
| Pain, Crushing | 3231 | HOXD1 |
| Pain, Crushing | 551 | AVP |
| Pain, Crushing | 3082 | HGF |
| Pain, Crushing | 4881 | NPR1 |
| Pain, Crushing | 4879 | NPPB |
| Pain, Crushing | 213 | ALB |
| Pain, Crushing | 7200 | TRH |
| Pain, Crushing | 1437 | CSF2 |
| Pain, Crushing | 3440 | IFNA2 |
| Pain, Crushing | 4986 | OPRK1 |
| Pain, Crushing | 7432 | VIP |
| Widespread Chronic Pain/ Nerve Pain | 6335 | SCN9A |
| Pain, Postoperative | 5243 | ABCB1 |
| Pain, Postoperative | 1394 | CRHR1 |
| Nerve Pain | 4988 | OPRM1 |
| Nerve Pain | 7852 | CXCR4 |
| Nerve Pain | 1586 | CYP17A1 |
| Nerve Pain | 6336 | SCN10A |
| Nerve Pain | 51083 | GAL |
| Nerve Pain | 4914 | NTRK1 |
| Nerve Pain | 2674 | GFRA1 |
| Nerve Pain | 2915 | GRM5 |
| Nerve Pain | 3146 | HMGB1 |
| Nerve Pain | 177 | AGER |
| Nerve Pain | 3358 | HTR2C |
| Pain, Intractable | 3576 | CXCL8 |

**Table S2.** The targets of cancer related pain in Genecards

| Gene Symbol | score | Gene Symbol | score | Gene Symbol | score |
| --- | --- | --- | --- | --- | --- |
| IL6 | 46.43 | TNF | 43.02 | NTRK1 | 35.16 |
| IL10 | 35.09 | IL1B | 33.77 | TP53 | 32.97 |
| IFNG | 28.35 | TLR4 | 28.05 | TGFB1 | 27.35 |
| PTGS2 | 26.72 | ESR1 | 25.57 | PTEN | 25.54 |
| CDKN2A | 25.52 | CCL2 | 25.48 | CDH1 | 25.45 |
| INS | 25.3 | EGFR | 24.41 | AKT1 | 24.39 |
| CCR6 | 24.2 | CCND1 | 24.03 | CTNNB1 | 23.93 |
| MMP2 | 23.89 | STAT3 | 22.87 | MIR21 | 22.83 |
| FAS | 21.93 | TNFRSF1A | 21.84 | PIK3CA | 21.75 |
| KRAS | 21.49 | MYC | 21.46 | ERBB2 | 21.23 |
| KIT | 21.1 | CRP | 21 | HRAS | 20.87 |
| BRCA1 | 20.85 | SPP1 | 20.75 | CALCA | 20.69 |
| EGF | 20.36 | MTOR | 20.26 | ICAM1 | 20.16 |
| MPO | 19.86 | APC | 19.78 | H19 | 19.76 |
| CDKN1A | 19.73 | MLH1 | 19.71 | TLR2 | 19.68 |
| MIR155 | 19.28 | MIR146A | 19.25 | RB1 | 19.22 |
| BRCA2 | 19.13 | SRC | 19.08 | CXCL8 | 18.99 |
| MET | 18.99 | CDK4 | 18.78 | IGF2 | 18.7 |
| MIR145 | 18.7 | FOXP3 | 18.68 | CAV1 | 18.65 |
| NOTCH1 | 18.6 | APOE | 18.59 | MIR17 | 18.53 |
| BRAF | 18.53 | ALB | 18.5 | RET | 18.4 |
| MIR125A | 18.39 | VDR | 18.34 | MIR142 | 18.23 |
| SMAD4 | 18.14 | CDKN1B | 18.1 | FASLG | 17.98 |
| CASP8 | 17.85 | ATM | 17.79 | MIF | 17.78 |
| MIR223 | 17.75 | MIR126 | 17.75 | MIR34A | 17.7 |
| PPARG | 17.67 | FLT1 | 17.52 | JAK2 | 17.5 |
| HLA-A | 17.47 | CTLA4 | 17.38 | CYP19A1 | 17.37 |
| PLAU | 17.3 | TGFBR2 | 17.29 | NOD2 | 17.27 |
| CDKN2B | 17.18 | MIR150 | 17.17 | VEGFA | 17.13 |
| MSH2 | 16.99 | DNMT1 | 16.96 | MIR143 | 16.96 |
| MMP9 | 16.94 | GNAS | 16.81 | MIR29A | 16.79 |
| HLA-DQB1 | 16.79 | FGFR3 | 16.67 | IL2 | 16.66 |
| TYMP | 16.54 | TGFB2 | 16.51 | TERT | 16.39 |
| MIR141 | 16.23 | MIR140 | 16.2 | MIR221 | 16.19 |
| BAX | 16.16 | HSPB1 | 16.06 | MIR200A | 16.03 |
| MIR27A | 16.03 | COMT | 16.02 | MIR31 | 15.97 |
| NOS2 | 15.93 | MMP14 | 15.89 | OPRM1 | 15.84 |
| SLC2A1 | 15.79 | ENG | 15.72 | TGFBR1 | 15.71 |
| NFE2L2 | 15.6 | EDNRA | 15.52 | PDGFRA | 15.52 |
| MIR214 | 15.52 | ABCG2 | 15.47 | BMP2 | 15.39 |
| SMAD3 | 15.38 | WT1 | 15.35 | MIR424 | 15.31 |
| PLG | 15.29 | ABCB1 | 15.24 | MMP13 | 15.23 |
| NRAS | 15.03 | MIR20A | 15.01 | LOX | 15 |
| MIR34C | 14.9 | AURKA | 14.86 | FGFR2 | 14.86 |
| TRPV1 | 14.79 | ABCC2 | 14.79 | STK11 | 14.76 |
| NF1 | 14.72 | TNFRSF10B | 14.68 | BDNF | 14.63 |
| MIR93 | 14.62 | AR | 14.55 | RELA | 14.38 |
| TEK | 14.37 | MIR200B | 14.35 | IL17A | 14.35 |
| ZEB1 | 14.34 | MIR10B | 14.33 | MIR127 | 14.32 |
| MIRLET7B | 14.32 | MAP2K1 | 14.3 | MAPK1 | 14.28 |
| PRKN | 14.24 | FOS | 14.2 | EDNRB | 14.13 |
| RAF1 | 14.09 | MIR132 | 14.08 | MIR182 | 14.05 |
| CCN2 | 14.04 | MIR29C | 14.02 | MIR195 | 14 |
| EPCAM | 13.98 | THBS2 | 13.98 | MIR25 | 13.86 |
| TGFB3 | 13.84 | NGF | 13.83 | CALR | 13.82 |
| SQSTM1 | 13.79 | PDCD1 | 13.77 | MIR200C | 13.75 |
| MIR100 | 13.69 | MIR224 | 13.66 | MIR106B | 13.58 |
| FHIT | 13.56 | MSH6 | 13.53 | IL1A | 13.51 |
| MIR205 | 13.45 | TOP2A | 13.44 | LRP5 | 13.36 |
| LEP | 13.36 | PRSS1 | 13.28 | SNAI2 | 13.26 |
| RAD51 | 13.25 | SPINK1 | 13.24 | TP63 | 13.22 |
| NOTCH3 | 13.19 | MIR22 | 13.18 | CD46 | 13.15 |
| ERBB4 | 13.13 | CXCR4 | 13.12 | MIRLET7A1 | 13.08 |
| TYMS | 12.98 | IL1RN | 12.98 | TLR5 | 12.97 |
| DDIT3 | 12.96 | MIR483 | 12.93 | CASP3 | 12.91 |
| MIR15A | 12.88 | EDN1 | 12.88 | CHEK2 | 12.85 |
| MIR181A1 | 12.84 | MIR183 | 12.81 | MIR144 | 12.74 |
| IGF1 | 12.71 | P2RX7 | 12.67 | ERCC6 | 12.67 |
| MIR222 | 12.66 | NKX2-1 | 12.56 | DKK1 | 12.56 |
| PPM1D | 12.54 | PMS2 | 12.51 | ABL1 | 12.48 |
| MIR210 | 12.48 | IDH1 | 12.48 | MIR23B | 12.43 |
| CASR | 12.42 | IL4 | 12.38 | JUN | 12.37 |
| MIRLET7D | 12.34 | MIR486-1 | 12.33 | CSF3 | 12.27 |
| TYR | 12.26 | ALK | 12.19 | MIR29B1 | 12.15 |
| MIR30E | 12.02 | MIR10A | 12 | MIR335 | 11.99 |
| MIR23A | 11.9 | MMP3 | 11.9 | DNMT3A | 11.89 |
| MIR146B | 11.83 | MT-CO1 | 11.78 | MIR19A | 11.77 |
| MTHFR | 11.69 | SLC6A4 | 11.66 | MIR18A | 11.64 |
| BIRC3 | 11.59 | FGF2 | 11.55 | MAPK8 | 11.52 |
| GSTP1 | 11.52 | ABCA1 | 11.42 | ETS1 | 11.41 |
| EP300 | 11.38 | PTGS1 | 11.34 | MIR148A | 11.32 |
| KLK3 | 11.31 | MIR15B | 11.31 | CHEK1 | 11.3 |
| SOX10 | 11.28 | DPYD | 11.22 | ITGB3 | 11.2 |
| MEFV | 11.11 | MIR373 | 11.1 | PGR | 11.08 |
| CD4 | 11.06 | HIF1A | 11.06 | IL13 | 11.05 |
| MDM2 | 11.04 | SEPTIN9 | 11.02 | TIMP1 | 11 |
| SERPINE1 | 11 | MIRLET7C | 10.99 | F2 | 10.91 |
| ATP7B | 10.89 | CCL5 | 10.87 | PLA2G2A | 10.86 |
| BCL2 | 10.85 | MIR342 | 10.8 | ACE | 10.76 |
| RARA | 10.75 | MAPK14 | 10.74 | MMP1 | 10.68 |
| NFKB1 | 10.68 | MIR203A | 10.65 | MITF | 10.63 |
| H2AC18 | 10.62 | MIR106A | 10.59 | MAPK3 | 10.58 |
| WNT1 | 10.57 | GSTM1 | 10.57 | KCNQ1OT1 | 10.55 |
| MIRLET7E | 10.51 | GPC3 | 10.51 | IRF1 | 10.5 |
| MIR331 | 10.48 | MIR204 | 10.45 | MYCN | 10.43 |
| MIRLET7G | 10.42 | CASP9 | 10.33 | HGF | 10.33 |
| MIR192 | 10.31 | DICER1 | 10.29 | BIRC5 | 10.28 |
| MAP2K2 | 10.27 | MRE11 | 10.26 | VIM | 10.26 |
| AXIN1 | 10.24 | ANXA5 | 10.21 | CDKN2C | 10.2 |
| IFNA1 | 10.2 | SETD2 | 10.18 | TCF4 | 10.17 |
| MIR320A | 10.16 | MIRLET7I | 10.14 | CTNNA1 | 10.14 |
| HMGA2 | 10.14 | CDKN3 | 10.12 | MIR96 | 10.12 |
| ODC1 | 10.1 | EZH2 | 10.08 | IGF1R | 10.07 |
| CD274 | 10.06 | MIR196B | 10.05 | BCL2L1 | 10.04 |
| IGFBP3 | 10.02 | MMP7 | 10.02 | MUC16 | 9.99 |
| BRIP1 | 9.97 | CD8A | 9.94 | SELE | 9.94 |
| FLCN | 9.91 | HLA-B | 9.9 | MIR451A | 9.88 |
| MUC1 | 9.85 | MIR181B1 | 9.83 | HNF1B | 9.79 |
| MIR199A1 | 9.76 | HPGD | 9.69 | CSF2 | 9.68 |
| OGG1 | 9.68 | IGF2R | 9.64 | KDR | 9.63 |
| IL6R | 9.63 | TIMP3 | 9.62 | LIFR | 9.58 |
| XRCC2 | 9.57 | KRT7 | 9.55 | ICOSLG | 9.52 |
| MIR34B | 9.52 | GATA4 | 9.5 | HP | 9.49 |
| DCC | 9.49 | MIR26A1 | 9.48 | NBN | 9.47 |
| PALB2 | 9.47 | MIR191 | 9.45 | CXCL12 | 9.45 |
| AXIN2 | 9.44 | MGMT | 9.41 | MIR199B | 9.41 |
| TNFSF10 | 9.37 | WNT3A | 9.35 | KRT17 | 9.32 |
| ESR2 | 9.28 | FN1 | 9.28 | PMS1 | 9.26 |
| VCAM1 | 9.26 | MYLK | 9.23 | EDN3 | 9.22 |
| TLR9 | 9.22 | OPRK1 | 9.21 | MIAT | 9.19 |
| PTK2 | 9.18 | SOD2 | 9.15 | F5 | 9.14 |
| HSP90AA1 | 9.09 | CNR1 | 9.08 | TNFSF11 | 9.07 |
| UBE3A | 9.05 | CD44 | 9.03 | PARP1 | 9.03 |
| MECOM | 9.03 | CD40 | 9.02 | MTR | 9.02 |
| RARB | 9 | EPHB2 | 8.98 | MIR30A | 8.96 |
| LGALS3 | 8.95 | BUB1B | 8.95 | NTN1 | 8.94 |
| NOS3 | 8.93 | MIR152 | 8.91 | WWOX | 8.9 |
| MIR184 | 8.9 | MIR128-2 | 8.9 | HMGB1 | 8.89 |
| TACR1 | 8.87 | GAPDH | 8.86 | BCL10 | 8.83 |
| MIR32 | 8.83 | TFG | 8.79 | NLRP3 | 8.78 |
| GNRH1 | 8.77 | PDGFRB | 8.76 | KLF6 | 8.76 |
| HOTAIR | 8.76 | CDH2 | 8.74 | CYP3A4 | 8.74 |
| TERC | 8.74 | SELP | 8.72 | ETV4 | 8.7 |
| PCNA | 8.7 | MALAT1 | 8.7 | BMP6 | 8.69 |
| CXCL1 | 8.67 | MIR9-1 | 8.65 | THBS1 | 8.64 |
| TGFA | 8.63 | TP73 | 8.62 | MIR375 | 8.61 |
| KRT19 | 8.61 | CEACAM5 | 8.58 | CYCS | 8.58 |
| CAT | 8.58 | AFP | 8.58 | POMC | 8.57 |
| WRN | 8.55 | SYNE1 | 8.54 | TSHR | 8.53 |
| STAT1 | 8.52 | CDK1 | 8.52 | CD40LG | 8.5 |
| ST14 | 8.49 | XRCC1 | 8.48 | COL2A1 | 8.48 |
| BARD1 | 8.38 | CCNA2 | 8.35 | MIR133B | 8.35 |
| XRCC3 | 8.33 | CSF1 | 8.33 | FSHR | 8.33 |
| CLU | 8.32 | TNFRSF10A | 8.32 | RASSF1 | 8.31 |
| CDKN2B-AS1 | 8.29 | PRL | 8.28 | MIR107 | 8.27 |
| MCL1 | 8.26 | IFNA2 | 8.25 | SOD1 | 8.24 |
| MUTYH | 8.21 | MIR196A1 | 8.19 | SP1 | 8.19 |
| MIR181A2 | 8.17 | HSPD1 | 8.17 | XIAP | 8.16 |
| CDK2 | 8.15 | HSPA4 | 8.13 | KRT20 | 8.12 |
| CREB1 | 8.12 | MEG3 | 8.12 | IL3 | 8.08 |
| SNAI1 | 8.08 | TIMP2 | 8.07 | COL1A1 | 8.04 |
| CTSD | 8.03 | TCF3 | 8.03 | GAST | 8.01 |
| BMP4 | 8.01 | PHB | 8.01 | MEN1 | 8 |
| E2F1 | 7.99 | VEGFC | 7.98 | GAS5 | 7.97 |
| HDC | 7.94 | MFAP5 | 7.88 | KRT18 | 7.87 |
| MT-CO2 | 7.87 | KITLG | 7.84 | ALPP | 7.84 |
| NME1 | 7.82 | CYP1A2 | 7.8 | F3 | 7.8 |
| FSCN1 | 7.79 | MME | 7.78 | PLAUR | 7.78 |
| SMAD2 | 7.76 | PPP2R1B | 7.75 | MIRLET7A3 | 7.75 |
| TOP1 | 7.75 | IL7 | 7.73 | POLE | 7.71 |
| PRKCA | 7.71 | GUCY2C | 7.67 | CDK6 | 7.64 |
| SLCO1B3 | 7.64 | CFTR | 7.62 | GDF15 | 7.62 |
| SIRT1 | 7.6 | PON1 | 7.6 | SPARC | 7.58 |
| MB | 7.57 | NFKBIA | 7.57 | NEAT1 | 7.56 |
| GDNF | 7.55 | MIR376A1 | 7.53 | MIR206 | 7.5 |
| CYP1A1 | 7.49 | RHOA | 7.49 | BECN1 | 7.48 |
| CYP2E1 | 7.46 | ELANE | 7.45 | NQO1 | 7.44 |
| MIR377 | 7.44 | RAD51D | 7.44 | IGFBP2 | 7.43 |
| HSPA1A | 7.42 | CCAT1 | 7.42 | CA9 | 7.41 |
| CCNB1 | 7.4 | ADIPOQ | 7.39 | PIK3CG | 7.39 |
| ROS1 | 7.39 | JAK1 | 7.39 | ITGB1 | 7.39 |
| TLR7 | 7.37 | ECRG4 | 7.37 | CCND2 | 7.36 |
| TLR3 | 7.36 | TTR | 7.36 | TJP1 | 7.35 |
| CYP1B1 | 7.33 | UCA1 | 7.31 | LGALS1 | 7.3 |
| ABCC1 | 7.29 | VTN | 7.26 | SST | 7.26 |
| CD80 | 7.25 | DHFR | 7.25 | CYP2D6 | 7.24 |
| PECAM1 | 7.24 | APAF1 | 7.22 | MIR24-2 | 7.19 |
| PIK3R1 | 7.19 | CFLAR | 7.19 | PVT1 | 7.19 |
| RAD51C | 7.17 | DACT1 | 7.17 | SOX2 | 7.16 |
| G6PD | 7.15 | CDX2 | 7.15 | CCR5 | 7.12 |
| CD34 | 7.12 | APOB | 7.12 | TWIST1 | 7.11 |
| DAPK1 | 7.11 | HDAC9 | 7.1 | BMI1 | 7.09 |
| TUG1 | 7.09 | CTSB | 7.08 | CSF1R | 7.08 |
| ERBB3 | 7.07 | MIR139 | 7.07 | TH | 7.05 |
| MIR328 | 7.05 | DLC1 | 7.03 | ERCC2 | 7.01 |
| GJA1 | 7 | SLPI | 7 | CYP2C9 | 6.99 |
| POU5F1 | 6.98 | MAP3K1 | 6.98 | MIR193A | 6.97 |
| MIR429 | 6.95 | FGFR1 | 6.93 | CARS2 | 6.92 |
| CD36 | 6.92 | BUB1 | 6.92 | EGR1 | 6.91 |
| TNNI3 | 6.9 | HFE | 6.9 | AREG | 6.89 |
| TFRC | 6.89 | CCND3 | 6.88 | PROM1 | 6.88 |
| MKI67 | 6.87 | MIR124-1 | 6.87 | CALB2 | 6.86 |
| TNFRSF11B | 6.86 | MIR101-1 | 6.85 | TGM2 | 6.85 |
| PTH | 6.83 | PDYN | 6.81 | U2AF1 | 6.81 |
| MYO5A | 6.81 | ANGPT1 | 6.78 | MIR137 | 6.77 |
| ATF3 | 6.77 | CASP7 | 6.77 | NAT2 | 6.76 |
| MIR99A | 6.76 | DCN | 6.73 | CTNND1 | 6.72 |
| MIR149 | 6.71 | FOXO3 | 6.7 | HSPA5 | 6.69 |
| ERCC1 | 6.68 | GADD45A | 6.67 | IL18 | 6.67 |
| HBEGF | 6.65 | LMNA | 6.64 | GCLC | 6.63 |
| MIR376B | 6.63 | PMP22 | 6.61 | GRP | 6.61 |
| PAX8 | 6.6 | AKT2 | 6.6 | MLF1 | 6.6 |
| ADORA1 | 6.59 | FOXM1 | 6.58 | IL1R1 | 6.58 |
| KRT8 | 6.58 | LTA | 6.58 | NTS | 6.58 |
| APEX1 | 6.57 | HTR3A | 6.56 | PLK1 | 6.56 |
| MUC5AC | 6.56 | CASP2 | 6.55 | MAP2K4 | 6.54 |
| MFN2 | 6.51 | TRIM28 | 6.5 | LCN2 | 6.5 |
| LOC110806262 | 6.48 | DPH1 | 6.47 | MIR215 | 6.47 |
| MIR2861 | 6.47 | SEMA4A | 6.47 | HMBS | 6.44 |
| NRG1 | 6.44 | IL2RB | 6.44 | RPS6KB1 | 6.44 |
| VHL | 6.43 | SPRY4-IT1 | 6.42 | MIR24-1 | 6.42 |
| IRS1 | 6.41 | MLH3 | 6.41 | CACNA1G | 6.4 |
| VEGFD | 6.4 | SDHB | 6.39 | SPIN1 | 6.38 |
| KRT14 | 6.37 | EZR | 6.37 | GPR68 | 6.35 |
| HDAC1 | 6.34 | CCKBR | 6.34 | SOCS3 | 6.33 |
| FLT4 | 6.33 | CYP17A1 | 6.33 | MMP12 | 6.33 |
| LPL | 6.32 | ELAVL1 | 6.31 | RRAS2 | 6.31 |
| PTPN12 | 6.31 | ABRAXAS1 | 6.31 | ANXA2 | 6.3 |
| RAD54B | 6.3 | MIR337 | 6.29 | CCAT2 | 6.2 |
| DMD | 6.17 | SKP2 | 6.16 | DIABLO | 6.14 |
| TPO | 6.14 | ITGA2 | 6.14 | GNRHR | 6.13 |
| UGT1A1 | 6.11 | CDC42 | 6.1 | MIR186 | 6.09 |
| DRD2 | 6.08 | GRB2 | 6.08 | LIF | 6.06 |
| MIR372 | 6.06 | MIR432 | 6.06 | FGF23 | 6.06 |
| NF2 | 6.05 | STAT4 | 6.05 | FCGR3A | 6.03 |
| BCL2L11 | 6.02 | MIR130B | 6.02 | SOX9 | 6.02 |
| EIF4E | 6.01 | CD86 | 6 | FASN | 5.99 |
| PLCG1 | 5.99 | RETREG1 | 5.98 | DNMT3B | 5.98 |
| ANGPT2 | 5.98 | ITGB4 | 5.97 | TNFRSF11A | 5.97 |
| HPRT1 | 5.97 | ARID1A | 5.97 | TKT | 5.96 |
| PTPRJ | 5.96 | WNT2B | 5.96 | FANCM | 5.96 |
| PDGFRL | 5.96 | OVCAS1 | 5.96 | LPAR1 | 5.95 |
| OCA2 | 5.94 | SERPINC1 | 5.94 | SNHG16 | 5.93 |
| COX5A | 5.92 | SMARCB1 | 5.92 | CD82 | 5.91 |
| TLR1 | 5.91 | GRIN2A | 5.91 | SERPINB5 | 5.9 |
| SNCA | 5.89 | PRKAR1A | 5.88 | SERPINA1 | 5.88 |
| PTPN11 | 5.88 | EEF1A1 | 5.87 | RECK | 5.86 |
| MMP11 | 5.86 | F2R | 5.86 | FZD6 | 5.86 |
| MIR338 | 5.84 | COL11A1 | 5.83 | SOCS1 | 5.83 |
| FBXW7 | 5.83 | EPHA2 | 5.83 | ILK | 5.82 |
| MIR125B1 | 5.81 | TG | 5.8 | CRNDE | 5.8 |
| PROS1 | 5.79 | PAEP | 5.79 | THPO | 5.78 |
| CTSK | 5.78 | MUC2 | 5.78 | IFI27 | 5.78 |
| XIST | 5.78 | FABP2 | 5.76 | EIF4EBP1 | 5.76 |
| PDGFB | 5.76 | OPCML | 5.75 | MIR376C | 5.75 |
| MIR495 | 5.75 | HULC | 5.74 | MIR542 | 5.74 |
| NRP1 | 5.74 | CD59 | 5.72 | XRCC6 | 5.69 |
| XRCC5 | 5.65 | MECP2 | 5.65 | ZFAS1 | 5.65 |
| AKT3 | 5.64 | MTA1 | 5.63 | FUS | 5.63 |
| CDH5 | 5.63 | BAK1 | 5.63 | CXCR2 | 5.62 |
| KLF4 | 5.61 | TLR6 | 5.61 | AMBP | 5.61 |
| PANDAR | 5.6 | S100A4 | 5.59 | APOA1 | 5.59 |
| TSPO | 5.58 | SDHD | 5.58 | CTSL | 5.57 |
| PRDM10 | 5.57 | HOXA11-AS | 5.56 | S100A8 | 5.56 |
| SMAD7 | 5.55 | MIR296 | 5.55 | CDC25C | 5.55 |
| VCP | 5.55 | EPO | 5.51 | LGALS3BP | 5.51 |
| MPL | 5.51 | CST3 | 5.5 | ZEB2 | 5.49 |
| MAPT | 5.49 | GRN | 5.48 | GSK3B | 5.47 |
| PRNP | 5.47 | DES | 5.47 | TSC2 | 5.46 |
| PSEN1 | 5.45 | MUC4 | 5.45 | KLRK1 | 5.44 |
| BAD | 5.44 | ACTC1 | 5.43 | MCC | 5.42 |
| BDKRB1 | 5.42 | LEF1 | 5.42 | PRKD1 | 5.4 |
| PIK3R2 | 5.4 | MT-CYB | 5.39 | RAC1 | 5.39 |
| BGLAP | 5.38 | KLK6 | 5.37 | BCYRN1 | 5.37 |
| PCAT1 | 5.37 | FH | 5.37 | CCL3 | 5.36 |
| PTGER2 | 5.36 | E2F3 | 5.36 | JUP | 5.36 |
| TCF7L2 | 5.35 | CTAG1B | 5.34 | CFH | 5.34 |
| CD24 | 5.34 | MIR574 | 5.33 | MIR103A1 | 5.33 |
| GLI1 | 5.3 | CDC73 | 5.3 | TNFRSF1B | 5.29 |
| TSG101 | 5.28 | TSC1 | 5.26 | NNT-AS1 | 5.26 |
| PTCH1 | 5.26 | FGF7 | 5.25 | BSG | 5.25 |
| GZMB | 5.25 | WNT5A | 5.25 | SDHA | 5.24 |
| ANPEP | 5.24 | TET2 | 5.24 | CEACAM3 | 5.23 |
| HSPA8 | 5.23 | TLR8 | 5.22 | FOLH1 | 5.22 |
| MIR361 | 5.22 | TFF1 | 5.22 | CACNA1H | 5.21 |
| WIF1 | 5.21 | PIK3R3 | 5.2 | PIK3CB | 5.19 |
| TLR10 | 5.19 | MIR27B | 5.18 | TXN | 5.18 |
| NGFR | 5.17 | DOCK8 | 5.17 | NPPB | 5.17 |
| LDHA | 5.16 | CASC2 | 5.16 | LINC-ROR | 5.16 |
| CCNE1 | 5.16 | COL1A2 | 5.16 | IL1RAPL2 | 5.15 |
| HAGLR | 5.13 | SDHC | 5.13 | NDRG1 | 5.13 |
| MIR92A1 | 5.13 | STAT5B | 5.12 | NPM1 | 5.12 |
| NOS1 | 5.12 | AFAP1-AS1 | 5.11 | FST | 5.11 |
| SERPINB2 | 5.11 | BNIP3 | 5.11 | FOSL1 | 5.11 |
| SHC1 | 5.11 | NANOG | 5.1 | MIR135A1 | 5.1 |
| L1CAM | 5.09 | MIR532 | 5.09 | YBX1 | 5.08 |
| HNRNPA2B1 | 5.08 | CHKB | 5.07 | IL2RA | 5.06 |
| RUNX3 | 5.04 | EPHX1 | 5.04 | PXN | 5.04 |
| FGF1 | 5.03 | SERPINB3 | 5.03 | ALOX12 | 5.01 |
| AGTR1 | 5 | SOX2-OT | 4.99 | PTTG1 | 4.98 |
| TP53COR1 | 4.98 | GUSB | 4.98 | CRCS11 | 4.98 |
| CRCS2 | 4.98 | CRCS5 | 4.98 | CRCS6 | 4.98 |
| CRCS9 | 4.98 | CRCS7 | 4.98 | CRCS8 | 4.98 |
| EPOR | 4.98 | CCL22 | 4.97 | KLK4 | 4.95 |
| DANCR | 4.94 | GHRL | 4.94 | HLA-G | 4.94 |
| GRIN2B | 4.93 | EPHB4 | 4.92 | MIR675 | 4.91 |
| KLK10 | 4.91 | BIRC2 | 4.9 | MSLN | 4.9 |
| CD9 | 4.89 | S100A9 | 4.89 | SNHG1 | 4.89 |
| ITGAV | 4.89 | GCRG224 | 4.88 | B2M | 4.87 |
| IL12B | 4.87 | ATP7A | 4.86 | GRPR | 4.86 |
| NTRK2 | 4.86 | CDH13 | 4.85 | CDC25A | 4.85 |
| CEACAM6 | 4.85 | CXCL10 | 4.84 | LCK | 4.84 |
| FURIN | 4.84 | PAX3 | 4.84 | PTPA | 4.84 |
| XPA | 4.84 | MAX | 4.84 | LRP6 | 4.83 |
| PTGER4 | 4.83 | HOTTIP | 4.82 | KLK8 | 4.81 |
| IL16 | 4.79 | DUSP1 | 4.78 | HSD17B1 | 4.78 |
| HSP90B1 | 4.78 | IDH2 | 4.78 | FLVCR1 | 4.75 |
| EWSR1 | 4.75 | CLDN7 | 4.75 | MVP | 4.75 |
| NCOA3 | 4.74 | CD247 | 4.74 | TGFBI | 4.72 |
| DNM2 | 4.69 | RNY1 | 4.69 | RNY3 | 4.69 |
| COL17A1 | 4.66 | VWF | 4.66 | FUT4 | 4.66 |
| PTPN13 | 4.66 | EBAG9 | 4.66 | KCNA1 | 4.66 |
| APOH | 4.64 | CDK5 | 4.64 | ITGAM | 4.63 |
| HMOX1 | 4.62 | BANCR | 4.61 | CCT5 | 4.6 |
| PTK2B | 4.59 | IKZF1 | 4.59 | GPER1 | 4.58 |
| SAT1 | 4.58 | SERPINA3 | 4.58 | HDAC2 | 4.58 |
| DCTN1 | 4.57 | AMACR | 4.57 | PDPN | 4.55 |
| MSH3 | 4.55 | FADD | 4.54 | MYH11 | 4.54 |
| KRT10 | 4.53 | MIR30C1 | 4.53 | MAOA | 4.53 |
| GH1 | 4.53 | EEF1A2 | 4.52 | MAP2K7 | 4.51 |
| MIR494 | 4.51 | AIFM1 | 4.51 | CIITA | 4.5 |
| TRPM8 | 4.49 | EPHA3 | 4.49 | RAD51B | 4.49 |
| YAP1 | 4.47 | BMPR1A | 4.46 | MYD88 | 4.46 |
| PKM | 4.46 | PENK | 4.45 | CYP24A1 | 4.45 |
| INHA | 4.45 | BCAR4 | 4.44 | KLK7 | 4.44 |
| HNF4A | 4.43 | CP | 4.43 | CLDN4 | 4.43 |
| SPINK7 | 4.43 | STAT6 | 4.42 | CYTOR | 4.42 |
| TCF7 | 4.42 | IRF5 | 4.41 | TMEM127 | 4.41 |
| CCL4 | 4.41 | MUC6 | 4.4 | IL12A | 4.4 |
| BCL3 | 4.4 | INHBA | 4.39 | S100B | 4.39 |
| MC1R | 4.39 | SFRP1 | 4.39 | SNHG12 | 4.38 |
| TBX1 | 4.38 | HPSE | 4.38 | MIR26B | 4.38 |
| FZD8 | 4.37 | NAMPT | 4.37 | ALDH1A1 | 4.37 |
| TP53BP1 | 4.36 | SNCG | 4.36 | SDHAF2 | 4.36 |
| ATR | 4.35 | ATRX | 4.35 | SNHG20 | 4.34 |
| FIP1L1 | 4.31 | CUL1 | 4.31 | CADM1 | 4.31 |
| PLAT | 4.3 | HPN | 4.3 | MAPK9 | 4.29 |
| IL24 | 4.29 | TNFRSF10C | 4.29 | ALPL | 4.29 |
| E2F4 | 4.29 | LIMK1 | 4.29 | BMPR2 | 4.28 |
| MIR345 | 4.28 | BAP1 | 4.28 | BLACAT1 | 4.28 |
| NPTN-IT1 | 4.28 | SLC4A1 | 4.28 | ENO2 | 4.26 |
| MBD4 | 4.24 | AXL | 4.23 | E2F2 | 4.23 |
| MIR135B | 4.21 | MIR422A | 4.21 | BACE1-AS | 4.21 |
| SS18 | 4.21 | CCL11 | 4.21 | MBL2 | 4.2 |
| MIR134 | 4.2 | IL5 | 4.19 | TNFSF13B | 4.18 |
| FLT3 | 4.18 | HSPB2 | 4.18 | EIF4G1 | 4.17 |
| KLK5 | 4.16 | LRP1 | 4.16 | UGT1A6 | 4.16 |
| CASP5 | 4.16 | SSX1 | 4.15 | CNBP | 4.15 |
| BAG1 | 4.15 | TNFRSF10D | 4.15 | FLI1 | 4.15 |
| RUNX2 | 4.15 | ARAF | 4.15 | SULF1 | 4.14 |
| AMHR2 | 4.14 | LINC00472 | 4.14 | MNX1-AS1 | 4.14 |
| FLG | 4.14 | ALOX5 | 4.13 | HTRA1 | 4.13 |
| GATA1 | 4.12 | CDC6 | 4.12 | RUNX1T1 | 4.12 |
| NAT1 | 4.12 | BID | 4.12 | SPG7 | 4.11 |
| MAGEA1 | 4.11 | IL15 | 4.11 | BCOR | 4.1 |
| ZNF217 | 4.09 | CLDN1 | 4.09 | NOTCH2 | 4.09 |
| JAG1 | 4.09 | HNF1A-AS1 | 4.09 | MIR497 | 4.09 |
| MIR509-1 | 4.08 | F2RL3 | 4.08 | WFDC2 | 4.08 |
| GLI3 | 4.08 | GSN | 4.08 | TNFRSF9 | 4.05 |
| GPX2 | 4.05 | SPINT1 | 4.05 | LASP1 | 4.05 |
| TPX2 | 4.05 | LCOR | 4.03 | NORAD | 4.03 |
| DCK | 4.03 | ACVR2A | 4.02 | MIR498 | 4.02 |
| VIP | 4.02 | MIR218-1 | 4.02 | FLNA | 4.02 |
| SOST | 4.01 | KMT2C | 4 | FBLN1 | 4 |
| SH2B3 | 4 |  |  |  |  |

**Table S3.** The targets of cancer related pain in OMIM

| Cytogenetic location | Gene/Locus | Gene/Locus MIM number |  |
| --- | --- | --- | --- |
| 1p33 | PAINQTL1 | 618377 | |
| 1q23.1 | NTRK1, TRKA | 191315 | |
| 2q12.2 | C2orf40, ECRG4 | 611752 | |
| 2q24.3 | SCN9A, NENA, PN1, FEB3B, GEFSP7, SFNP, HSAN2D | 603415 | |
| 2q24.3 | SCN9A, NENA, PN1, FEB3B, GEFSP7, SFNP, HSAN2D | 603415 | |
| 2q24.3 | SCN9A, NENA, PN1, FEB3B, GEFSP7, SFNP, HSAN2D | 603415 | |
| 2q24.3 | SCN9A, NENA, PN1, FEB3B, GEFSP7, SFNP, HSAN2D | 603415 | |
| 2q24.3 | SCN9A, NENA, PN1, FEB3B, GEFSP7, SFNP, HSAN2D | 603415 | |
| 2q24.3 | SCN9A, NENA, PN1, FEB3B, GEFSP7, SFNP, HSAN2D | 603415 | |
| 2q24.3 | SCN9A, NENA, PN1, FEB3B, GEFSP7, SFNP, HSAN2D | 603415 | |
| 2q24.3 | SCN9A, NENA, PN1, FEB3B, GEFSP7, SFNP, HSAN2D | 603415 | |
| 3p22.2 | SCN10A, FEPS2 | 604427 | |
| 3p22.2 | SCN11A, HSAN7, FEPS3 | 604385 | |
| 3p22.2 | SCN11A, HSAN7, FEPS3 | 604385 | |
| 8q21.11 | TRPA1, ANKTM1, FEPS1 | 604775 | |
| 11q14 | GCRG224 | 610888 | |
| 17p13.3 | BCPR | 113721 | |

**Table S4.** All the GTF compounds

| Chinese name | Latin name | Mol ID | Active compound | OB (%) | DL |
| --- | --- | --- | --- | --- | --- |
| Ding Xiang (丁香) | Flos Caryophylli | MOL013219 | Strictosamide_qt | 76.3 | 0.76 |
|  |  | MOL001749 | ZINC03860434 | 43.59 | 0.35 |
|  |  | MOL000098 | Quercetin | 46.43 | 0.28 |
| Ding Xiang/Gan Jiang/Shan Cigu/Wei Linxian/Xian Mao (丁香/干姜/山慈菇/威灵仙/仙茅) | Flos Caryophylli/ Rhizoma Zingiberis/ Pseudobulbus Cremastrae Seu Pleiones/ Radix Clematidis/ Rhizoma Curculigins | MOL000358 | *β*-sitosterol | 36.91 | 0.75 |
| Ding Xiang/Xi Xin (丁香/细辛) | Flos Caryophylli/ Herba Asari | MOL000422 | Kaempferol | 41.88 | 0.24 |
| Ding Xiang/Shan Cigu/Wei Linxian/Xian Mao (丁香/山慈菇/威灵仙/仙茅) | Flos Caryophylli/ Pseudobulbus Cremastrae Seu Pleiones/ Radix Clematidis/ Rhizoma Curculigins | MOL000449 | Stigmasterol | 43.83 | 0.76 |
| Fu Zi (附子) | Radix Aconiti Lateralis Preparata | MOL002416 | Deoxyaconitine | 30.96 | 0.24 |
|  |  | MOL000538 | Hypaconitine | 31.39 | 0.26 |
|  |  | MOL002423 | Jesaconitine | 33.41 | 0.19 |
|  |  | MOL002410 | Benzoylnapelline | 34.06 | 0.53 |
|  |  | MOL002393 | Demethyldelavaine A | 34.52 | 0.18 |
|  |  | MOL002394 | Demethyldelavaine B | 34.52 | 0.18 |
|  |  | MOL002434 | Carnosifloside I_qt | 38.16 | 0.8 |
|  |  | MOL002406 | CID5316641 | 39.43 | 0.38 |
|  |  | MOL002211 | Eicosadienoic acid | 39.99 | 0.2 |
| Fu Zi (附子) | Radix Aconiti Lateralis Preparata | MOL002433 | CID6324887 | 41.52 | 0.22 |
|  |  | MOL002401 | Neokadsuranic acid B | 43.1 | 0.85 |
|  |  | MOL002392 | Deltoin | 46.69 | 0.37 |
|  |  | MOL002422 | Isotalatizidine | 50.82 | 0.73 |
|  |  | MOL002397 | Karakoline | 51.73 | 0.73 |
|  |  | MOL002415 | 6-Demethyldesoline | 51.87 | 0.66 |
|  |  | MOL002395 | Deoxyandrographolide | 56.3 | 0.31 |
|  |  | MOL002388 | Delphin_qt | 57.76 | 0.28 |
|  |  | MOL002398 | Karanjin | 69.56 | 0.34 |
|  |  | MOL002419 | (R)-Norcoclaurine | 82.54 | 0.21 |
|  |  | MOL002421 | Ignavine | 84.08 | 0.25 |
| Gan Jiang (干姜) | Rhizoma Zingiberis | MOL002464 | 1-Monolinolein | 37.18 | 0.3 |
|  |  | MOL002514 | Sexangularetin | 62.86 | 0.3 |
| Fu Zi/Gan Jiang (附子/干姜) | Radix Aconiti Lateralis Preparata/ Rhizoma Zingiberis | MOL000359 | Sitosterol | 36.91 | 0.75 |
| Gan Jiang/Xi xin (干姜/细辛) | Rhizoma Zingiberis/ Herba Asari | MOL002501 | SCHEMBL119969 | 62.52 | 0.31 |
| Shan Cigu (山慈菇) | Pseudobulbus Cremastrae Seu Pleiones | MOL007991 | CHEMBL254186 | 44.97 | 0.18 |
| Wei Linxian (威灵仙) | Radix Clematidis | MOL001663 | Epi-Oleanolic acid | 32.03 | 0.76 |
|  |  | MOL002372 | Spinacen | 33.55 | 0.42 |
|  |  | MOL005594 | ClematosideA'_qt | 37.51 | 0.76 |
|  |  | MOL005598 | Embinin | 33.91 | 0.73 |
|  |  | MOL005603 | Heptyl phthalate | 42.26 | 0.31 |
| Xi Xin (细辛) | Herba Asari | MOL012140 | Picrasidine D | 65.3 | 0.19 |
|  |  | MOL012141 | Caribine | 37.06 | 0.83 |
|  |  | MOL001460 | Cryptopin | 78.74 | 0.72 |
|  |  | MOL001558 | Sesamin | 56.55 | 0.83 |
|  |  | MOL002962 | 3-O-Methylviolanone | 48.23 | 0.33 |
|  |  | MOL009849 | ZINC05223929 | 31.57 | 0.83 |
| Xian Mao (仙茅) | Rhizoma Curculigins | MOL004137 | Yuccagenin | 12.1 | 0.79 |
|  |  | MOL004162 | Curculigenin A | 12.61 | 0.79 |
|  |  | MOL004150 | Curculigosaponin J_qt | 26.45 | 0.78 |
|  |  | MOL004164 | Curculigenin C | 29.85 | 0.78 |
|  |  | MOL004168 | Curculigol | 14.24 | 0.78 |
|  |  | MOL004144 | Curculigosaponin E_qt | 26.45 | 0.78 |
|  |  | MOL004123 | Curculigosaponin G_qt | 26.45 | 0.78 |
|  |  | MOL004158 | Curculigosaponin L_qt | 18.45 | 0.78 |
|  |  | MOL004163 | Curculigenin B | 9.88 | 0.78 |
|  |  | MOL004161 | Curculigosaponin M_qt | 29.85 | 0.78 |
|  |  | MOL003578 | Cycloartenol | 38.69 | 0.78 |
|  |  | MOL001607 | ZINC03982454 | 36.91 | 0.76 |
|  |  | MOL004165 | CID11503965 | 3.94 | 0.73 |
|  |  | MOL000396 | (+)-Syringaresinol | 3.29 | 0.72 |
|  |  | MOL004126 | Curculigoside | 14.89 | 0.71 |
|  |  | MOL004139 | Curculigine A | 13.9 | 0.7 |
| Xian Mao (仙茅) | Rhizoma Curculigins | MOL004124 | Curculigoside B | 7.8 | 0.67 |
|  |  | MOL004120 | Curculigin C | 18.24 | 0.66 |
|  |  | MOL000357 | Sitogluside | 20.63 | 0.62 |
|  |  | MOL004141 | Curculigine B | 20.82 | 0.62 |
|  |  | MOL004138 | Corchioside A | 12.77 | 0.52 |
|  |  | MOL004136 | Lycorine | 12.62 | 0.51 |
|  |  | MOL004145 | Curculigosaponin B | 10.34 | 0.51 |
|  |  | MOL004118 | 4-acetyl-2-methyoxy-5-methyltriacontane | 17.9 | 0.48 |
|  |  | MOL004143 | Curculigosaponin A | 7.32 | 0.39 |
|  |  | MOL004135 | NSC643978 | 29.24 | 0.33 |
| Quan Xie (全蝎) | Scorpio | MOL000987 | Cholesterol | 5.69 | 0.67 |
|  |  | MOL004082 | Stearin | 15.13 | 0.13 |
|  |  | MOL011455 | 20-hexadecanoylingenol | 32.7 | 0.65 |
|  |  | / | Bufotoxin | / | / |
|  |  | / | chlorotoxin | / | / |
|  |  | / | katsutoxin | / | / |

**Table S5.** Potential targets of GTF compounds downloaded from TCMSP database

| Mol ID | Active compound | Target | Gene symbol |
| --- | --- | --- | --- |
| MOL000098 | Quercetin | Prostaglandin G/H synthase 1 | PTGS1 |
|  |  | Androgen receptor | AR |
|  |  | Prostaglandin G/H synthase 2 | PTGS2 |
|  |  | Phosphatidylinositol-4,5-bisphosphate 3-kinase catalytic subunit, gamma isoform | PIK3CG |
|  |  | Nuclear receptor coactivator 2 | NCOA2 |
|  |  | Dipeptidyl peptidase IV | DPP4 |
|  |  | Aldose reductase | AKR1B1 |
|  |  | Trypsin-1 | PRSS1 |
|  |  | Thrombin | F2 |
|  |  | Potassium voltage-gated channel subfamily H member 2 | KCNH2 |
|  |  | Sodium channel protein type 5 subunit alpha | SCN5A |
|  |  | Coagulation factor Xa | F10 |
|  |  | Beta-2 adrenergic receptor | ADRB2 |
|  |  | Stromelysin-1 | MMP3 |
|  |  | Coagulation factor VII | F7 |
|  |  | Retinoic acid receptor RXR-alpha | RXRA |
|  |  | Acetylcholinesterase | ACHE |
|  |  | Gamma-aminobutyric acid receptor subunit alpha-1 | GABRA1 |
|  |  | Amine oxidase [flavin-containing] B | MAOB |
|  |  | Transcription factor p65 | RELA |
|  |  | Epidermal growth factor receptor | EGFR |
| Continue with the table below | | | |
| MOL000098 | Quercetin | RAC-alpha serine/threonine-protein kinase | AKT1 |
|  |  | Vascular endothelial growth factor A | VEGFA |
|  |  | G1/S-specific cyclin-D1 | CCND1 |
|  |  | Apoptosis regulator Bcl-2 | BCL2 |
|  |  | Bcl-2-like protein 1 | BCL2L1 |
|  |  | Proto-oncogene c-Fos | FOS |
|  |  | Cyclin-dependent kinase inhibitor 1 | CDKN1A |
|  |  | Eukaryotic translation initiation factor 6 | EIF6 |
|  |  | Apoptosis regulator BAX | BAX |
|  |  | Caspase-9 | CASP9 |
|  |  | Urokinase-type plasminogen activator | PLAU |
|  |  | 72 kDa type IV collagenase | MMP2 |
|  |  | Matrix metalloproteinase-9 | MMP9 |
|  |  | Mitogen-activated protein kinase 1 | MAPK1 |
|  |  | Interleukin-10 | IL10 |
|  |  | Pro-epidermal growth factor | EGF |
|  |  | Retinoblastoma-associated protein | RB1 |
|  |  | Tumor necrosis factor | TNF |
|  |  | Transcription factor AP-1 | JUN |
|  |  | Interleukin-6 | IL6 |
|  |  | Cyclin-dependent kinase inhibitor 2A, isoforms 1/2/3 | CDKN2A |
|  |  | Activator of 90 kDa heat shock protein ATPase homolog 1 | AHSA1 |
|  |  | Caspase-3 | CASP3 |
| MOL000098 | Quercetin | Cellular tumor antigen p53 | TP63 |
|  |  | ETS domain-containing protein Elk-1 | ELK1 |
|  |  | NF-kappa-B inhibitor alpha | NFKBIA |
|  |  | NADPH--cytochrome P450 reductase | POR |
|  |  | Ornithine decarboxylase | ODC1 |
|  |  | Xanthine dehydrogenase/oxidase | XDH |
|  |  | Caspase-8 | CASP8 |
|  |  | DNA topoisomerase 1 | TOP1 |
|  |  | RAF proto-oncogene serine/threonine-protein kinase | RAF1 |
|  |  | Superoxide dismutase [Cu-Zn] | SOD1 |
|  |  | Protein kinase C alpha type | PRKCA |
|  |  | Interstitial collagenase | MMP1 |
|  |  | Hypoxia-inducible factor 1-alpha | HIF1A |
|  |  | Signal transducer and activator of transcription 1-alpha/beta | STAT1 |
|  |  | Protein CBFA2T1 | RUNX1T1 |
|  |  | 78 kDa glucose-regulated protein | HSPA5 |
|  |  | Receptor tyrosine-protein kinase erbB-2 | ERBB2 |
|  |  | Peroxisome proliferator-activated receptor gamma | PPARG |
|  |  | Acetyl-CoA carboxylase 1 | ACACA |
|  |  | Heme oxygenase 1 | HMOX1 |
|  |  | Cytochrome P450 3A4 | CYP3A4 |
|  |  | Cytochrome P450 1A2 | CYP1A2 |
|  |  | Caveolin-1 | CAV1 |
| MOL000098 | Quercetin | Myc proto-oncogene protein | MYC |
|  |  | Tissue factor | F3 |
|  |  | Gap junction alpha-1 protein | GJA1 |
|  |  | Cytochrome P450 1A1 | CYP1A1 |
|  |  | Intercellular adhesion molecule 1 | ICAM1 |
|  |  | Interleukin-1 beta | IL1B |
|  |  | C-C motif chemokine 2 | CCL2 |
|  |  | E-selectin | SELE |
|  |  | Vascular cell adhesion protein 1 | VCAM1 |
|  |  | Prostaglandin E2 receptor EP3 subtype | PTGER3 |
|  |  | Interleukin-8 | CXCL8 |
|  |  | Protein kinase C beta type | PRKCB |
|  |  | Baculoviral IAP repeat-containing protein 5 | BIRC5 |
|  |  | Dual oxidase 2 | DUOX2 |
|  |  | Nitric oxide synthase, endothelial | NOS3 |
|  |  | Heat shock protein beta-1 | HSPB1 |
|  |  | Transforming growth factor beta-1 | TGFB1 |
|  |  | Estrogen sulfotransferase | SULT1E1 |
|  |  | Maltase-glucoamylase, intestinal | MGAM |
|  |  | Interleukin-2 | IL2 |
|  |  | Nuclear receptor subfamily 1 group I member 2 | NR1I2 |
|  |  | Cytochrome P450 1B1 | CYP1B1 |
|  |  | G2/mitotic-specific cyclin-B1 | CCNB1 |
|  |  | Tissue-type plasminogen activator | PLAT |
| MOL000098 | Quercetin | Thrombomodulin | THBD |
|  |  | Plasminogen activator inhibitor 1 | SERPINE1 |
|  |  | Collagen alpha-1(I) chain | COL1A1 |
|  |  | Interferon gamma | IFNG |
|  |  | Arachidonate 5-lipoxygenase | ALOX5 |
|  |  | Phosphatidylinositol-3,4,5-trisphosphate 3-phosphatase and dual-specificity protein phosphatase PTEN | PTEN |
|  |  | Interleukin-1 alpha | IL1A |
|  |  | Myeloperoxidase | MPO |
|  |  | DNA topoisomerase 2-alpha | TOP2A |
|  |  | Neutrophil cytosol factor 1 | NCF1 |
|  |  | ATP-binding cassette sub-family G member 2 | ABCG2 |
|  |  | Hyaluronan synthase 2 | HAS2 |
|  |  | Glutathione S-transferase P | GSTP1 |
|  |  | Nuclear factor erythroid 2-related factor 2 | NFE2L2 |
|  |  | NAD(P)H dehydrogenase [quinone] 1 | NQO1 |
|  |  | Poly [ADP-ribose] polymerase 1 | PARP1 |
|  |  | Aryl hydrocarbon receptor | AHR |
|  |  | 26S proteasome non-ATPase regulatory subunit 3 | PSMD3 |
|  |  | Solute carrier family 2, facilitated glucose transporter member 4 | SLC2A4 |
|  |  | Collagen alpha-1(III) chain | COL3A1 |
|  |  | DNA gyrase subunit B | GyrB |
|  |  | C-X-C motif chemokine 11 | CXCL11 |
| MOL000098 | Quercetin | C-X-C motif chemokine 2 | CXCL2 |
|  |  | DDB1- and CUL4-associated factor 5 | DCAF5 |
|  |  | Nuclear receptor subfamily 1 group I member 3 | NR1I3 |
|  |  | Serine/threonine-protein kinase Chk2 | CHEK2 |
|  |  | Insulin receptor | INSR |
|  |  | Claudin-4 | CLDN4 |
|  |  | Peroxisome proliferator-activated receptor alpha | PPARA |
|  |  | Peroxisome proliferator-activated receptor delta | PPARD |
|  |  | Heat shock factor protein 1 | HSF1 |
|  |  | C-reactive protein | CRP |
|  |  | C-X-C motif chemokine 10 | CXCL10 |
|  |  | Inhibitor of nuclear factor kappa-B kinase subunit alpha | CHUK |
|  |  | Osteopontin | SPP1 |
|  |  | Runt-related transcription factor 2 | RUNX2 |
|  |  | Ras association domain-containing protein 1 | RASSF1 |
|  |  | Transcription factor E2F1 | E2F1 |
|  |  | Transcription factor E2F2 | E2F2 |
|  |  | Prostatic acid phosphatase | ACPP |
|  |  | Cathepsin D | CTSD |
|  |  | Insulin-like growth factor-binding protein 3 | IGFBP3 |
|  |  | Insulin-like growth factor II | IGF2 |
|  |  | CD40 ligand | CD40LG |
| MOL000098 | Quercetin | Interferon regulatory factor 1 | IRF1 |
|  |  | Receptor tyrosine-protein kinase erbB-3 | ERBB3 |
|  |  | Serum paraoxonase/arylesterase 1 | PON1 |
|  |  | Type I iodothyronine deiodinase | DIO1 |
|  |  | Procollagen C-endopeptidase enhancer 1 | PCOLCE |
|  |  | Puromycin-sensitive aminopeptidase | NPEPPS |
|  |  | Hexokinase-2 | HK2 |
|  |  | Homeobox protein Nkx-3.1 | NKX3-1 |
|  |  | Ras GTPase-activating protein 1 | RASA1 |
|  |  | Glutathione S-transferase Mu 1 | GSTM1 |
|  |  | Glutathione S-transferase Mu 2 | GSTM2 |
| MOL000357 | Sitogluside | Progesterone receptor | PGR |
|  |  | Prostaglandin G/H synthase 1 | PTGS1 |
|  |  | Muscarinic acetylcholine receptor M3 | CHRM3 |
|  |  | Potassium voltage-gated channel subfamily H member 2 | KCNH2 |
|  |  | Muscarinic acetylcholine receptor M1 | CHRM1 |
|  |  | Sodium channel protein type 5 subunit alpha | SCN5A |
|  |  | Coagulation factor Xa | F10 |
|  |  | Prostaglandin G/H synthase 2 | PTGS2 |
|  |  | 5-hydroxytryptamine receptor 3A | HTR3A |
|  |  | Retinoic acid receptor RXR-alpha | RXRA |
|  |  | CGMP-inhibited 3',5'-cyclic phosphodiesterase A | PDE3A |
| MOL000357 | Sitogluside | Alpha-1B adrenergic receptor | ADRA1B |
|  |  | Beta-2 adrenergic receptor | ADRB2 |
|  |  | Alpha-1D adrenergic receptor | ADRA1D |
|  |  | Nuclear receptor coactivator 2 | NCOA2 |
| MOL000358 | *β-*sitosterol | Progesterone receptor | PGR |
|  |  | Nuclear receptor coactivator 2 | NCOA2 |
|  |  | Prostaglandin G/H synthase 1 | PTGS1 |
|  |  | Prostaglandin G/H synthase 2 | PTGS2 |
|  |  | Phosphatidylinositol-4,5-bisphosphate 3-kinase catalytic subunit, gamma isoform | PIK3CG |
|  |  | Potassium voltage-gated channel subfamily H member 2 | KCNH2 |
|  |  | Dopamine D1 receptor | DRD1 |
|  |  | Muscarinic acetylcholine receptor M3 | CHRM3 |
|  |  | Muscarinic acetylcholine receptor M1 | CHRM1 |
|  |  | Sodium channel protein type 5 subunit alpha | SCN5A |
|  |  | Gamma-aminobutyric-acid receptor alpha-2 subunit | GABRA2 |
|  |  | Muscarinic acetylcholine receptor M4 | CHRM4 |
|  |  | CGMP-inhibited 3',5'-cyclic phosphodiesterase A | PDE3A |
|  |  | 5-hydroxytryptamine 2A receptor | HTR2A |
|  |  | Gamma-aminobutyric-acid receptor alpha-5 subunit | GABRA5 |
|  |  | Alpha-1A adrenergic receptor | ADRA1A |
|  |  | Gamma-aminobutyric-acid receptor alpha-3 subunit | GABRA3 |
| MOL000358 | *β*-sitosterol | Muscarinic acetylcholine receptor M2 | CHRM2 |
|  |  | Alpha-1B adrenergic receptor | ADRA1B |
|  |  | Beta-2 adrenergic receptor | ADRB2 |
|  |  | Neuronal acetylcholine receptor subunit alpha-2 | CHRNA2 |
|  |  | Sodium-dependent serotonin transporter | SLC6A4 |
|  |  | Mu-type opioid receptor | OPRM1 |
|  |  | Gamma-aminobutyric acid receptor subunit alpha-1 | GABRA1 |
|  |  | Neuronal acetylcholine receptor protein, alpha-7 chain | CHRNA7 |
|  |  | Apoptosis regulator Bcl-2 | BCL2 |
|  |  | Apoptosis regulator BAX | BAX |
|  |  | Caspase-9 | CASP9 |
|  |  | Transcription factor AP-1 | JUN |
|  |  | Caspase-3 | CASP3 |
|  |  | Caspase-8 | CASP8 |
|  |  | Protein kinase C alpha type | PRKCA |
|  |  | Transforming growth factor beta-1 | TGFB1 |
|  |  | Serum paraoxonase/arylesterase 1 | PON1 |
|  |  | Microtubule-associated protein 2 | MAP2 |
|  |  | Progesterone receptor | PGR |
|  |  | Nuclear receptor coactivator 2 | NCOA2 |
|  |  | Mineralocorticoid receptor | NR3C2 |
| MOL000396 | (+)-Syringaresinol | Potassium voltage-gated channel subfamily H member 2 | KCNH2 |
| MOL000396 | (+)-Syringaresinol | Sodium channel protein type 5 subunit alpha | SCN5A |
|  |  | Coagulation factor Xa | F10 |
|  |  | Prostaglandin G/H synthase 2 | PTGS2 |
|  |  | Nuclear receptor coactivator 2 | NCOA2 |
| MOL000422 | Kaempferol | Nitric oxide synthase, inducible | NOS2 |
|  |  | Prostaglandin G/H synthase 1 | PTGS1 |
|  |  | Androgen receptor | AR |
|  |  | Peroxisome proliferator activated receptor gamma | PPARG |
|  |  | Prostaglandin G/H synthase 2 | PTGS2 |
|  |  | Phosphatidylinositol-4,5-bisphosphate 3-kinase catalytic subunit, gamma isoform | PIK3CG |
|  |  | Nuclear receptor coactivator 2 | NCOA2 |
|  |  | Dipeptidyl peptidase IV | DPP4 |
|  |  | Trypsin-1 | PRSS1 |
|  |  | Progesterone receptor | PGR |
|  |  | Thrombin | F2 |
|  |  | Muscarinic acetylcholine receptor M1 | CHRM1 |
|  |  | Nitric-oxide synthase, endothelial | NOS3 |
|  |  | Gamma-aminobutyric-acid receptor alpha-2 subunit | GABRA2 |
|  |  | Acetylcholinesterase | ACHE |
|  |  | Sodium-dependent noradrenaline transporter | SLC6A2 |
|  |  | Muscarinic acetylcholine receptor M2 | CHRM2 |
|  |  | Alpha-1B adrenergic receptor | ADRA1B |
| MOL000422 | Kaempferol | Gamma-aminobutyric acid receptor subunit alpha-1 | GABRA1 |
|  |  | Coagulation factor VII | F7 |
|  |  | Transcription factor p65 | RELA |
|  |  | Inhibitor of nuclear factor kappa-B kinase subunit beta | IKBKB |
|  |  | RAC-alpha serine/threonine-protein kinase | AKT1 |
|  |  | Apoptosis regulator Bcl-2 | BCL2 |
|  |  | Apoptosis regulator BAX | BAX |
|  |  | Tumor necrosis factor | TNF |
|  |  | Transcription factor AP-1 | JUN |
|  |  | Activator of 90 kDa heat shock protein ATPase homolog 1 | AHSA1 |
|  |  | Caspase-3 | CASP3 |
|  |  | Mitogen-activated protein kinase 8 | MAPK8 |
|  |  | Xanthine dehydrogenase/oxidase | XDH |
|  |  | Interstitial collagenase | MMP1 |
|  |  | Signal transducer and activator of transcription 1-alpha/beta | STAT1 |
|  |  | Heme oxygenase 1 | HMOX1 |
|  |  | Cytochrome P450 3A4 | CYP3A4 |
|  |  | Cytochrome P450 1A2 | CYP1A2 |
|  |  | Cytochrome P450 1A1 | CYP1A1 |
|  |  | Intercellular adhesion molecule 1 | ICAM1 |
|  |  | E-selectin | SELE |
|  |  | Vascular cell adhesion protein 1 | VCAM1 |
| MOL000422 | Kaempferol | Nuclear receptor subfamily 1 group I member 2 | NR1I2 |
|  |  | Cytochrome P450 1B1 | CYP1B1 |
|  |  | Arachidonate 5-lipoxygenase | ALOX5 |
|  |  | Hyaluronan synthase 2 | HAS2 |
|  |  | Glutathione S-transferase P | GSTP1 |
|  |  | Aryl hydrocarbon receptor | AHR |
|  |  | 26S proteasome non-ATPase regulatory subunit 3 | PSMD3 |
|  |  | Solute carrier family 2, facilitated glucose transporter member 4 | SLC2A4 |
|  |  | Nuclear receptor subfamily 1 group I member 3 | NR1I3 |
|  |  | Insulin receptor | INSR |
|  |  | Type I iodothyronine deiodinase | DIO1 |
|  |  | Serine/threonine-protein phosphatase 2B catalytic subunit alpha isoform | PPP3CA |
|  |  | Glutathione S-transferase Mu 1 | GSTM1 |
|  |  | Glutathione S-transferase Mu 2 | GSTM2 |
|  |  | Aldo-keto reductase family 1 member C3 | AKR1C3 |
|  |  | Antileukoproteinase | SLPI |
|  |  | Peroxisome proliferator-activated receptor gamma | PPARG |
| MOL000449 | Stigmasterol | Progesterone receptor | PGR |
|  |  | Mineralocorticoid receptor | NR3C2 |
|  |  | Nuclear receptor coactivator 2 | NCOA2 |
|  |  | Alcohol dehydrogenase 1C | ADH1C |
| MOL000449 | Stigmasterol | Ig gamma-1 chain C region | IGHG1 |
|  |  | Retinoic acid receptor RXR-alpha | RXRA |
|  |  | Nuclear receptor coactivator 1 | NCOA1 |
|  |  | Prostaglandin G/H synthase 1 | PTGS1 |
|  |  | Prostaglandin G/H synthase 2 | PTGS2 |
|  |  | Alpha-2A adrenergic receptor | ADRA2A |
|  |  | Sodium-dependent noradrenaline transporter | SLC6A2 |
|  |  | Sodium-dependent dopamine transporter | SLC6A3 |
|  |  | Beta-2 adrenergic receptor | ADRB2 |
|  |  | Aldose reductase | AKR1B1 |
|  |  | Urokinase-type plasminogen activator | PLAU |
|  |  | Leukotriene A-4 hydrolase | LTA4H |
|  |  | Amine oxidase [flavin-containing] B | MAOB |
|  |  | Amine oxidase [flavin-containing] A | MAOA |
|  |  | Chymotrypsinogen B | CTRB1 |
|  |  | Muscarinic acetylcholine receptor M3 | CHRM3 |
|  |  | Muscarinic acetylcholine receptor M1 | CHRM1 |
|  |  | Beta-1 adrenergic receptor | ADRB1 |
|  |  | Sodium channel protein type 5 subunit alpha | SCN5A |
|  |  | 5-hydroxytryptamine 2A receptor | HTR2A |
|  |  | Alpha-1A adrenergic receptor | ADRA1A |
|  |  | Gamma-aminobutyric-acid receptor alpha-3 subunit | GABRA3 |
|  |  | Muscarinic acetylcholine receptor M2 | CHRM2 |
| MOL000449 | Stigmasterol | Alpha-1B adrenergic receptor | ADRA1B |
|  |  | Gamma-aminobutyric acid receptor subunit alpha-1 | GABRA1 |
|  |  | Neuronal acetylcholine receptor protein, alpha-7 chain | CHRNA7 |
| MOL001460 | Cryptopin | Prostaglandin G/H synthase 1 | PTGS1 |
|  |  | Dopamine D1 receptor | DRD1 |
|  |  | Muscarinic acetylcholine receptor M3 | CHRM3 |
|  |  | Potassium voltage-gated channel subfamily H member 2 | KCNH2 |
|  |  | Muscarinic acetylcholine receptor M1 | CHRM1 |
|  |  | Sodium channel protein type 5 subunit alpha | SCN5A |
|  |  | Coagulation factor Xa | F10 |
|  |  | Muscarinic acetylcholine receptor M5 | CHRM5 |
|  |  | Prostaglandin G/H synthase 2 | PTGS2 |
|  |  | 5-hydroxytryptamine receptor 3A | HTR3A |
|  |  | Coagulation factor VII | F7 |
|  |  | Vascular endothelial growth factor receptor 2 | KDR |
|  |  | Muscarinic acetylcholine receptor M4 | CHRM4 |
|  |  | Delta-type opioid receptor | OPRD1 |
|  |  | CGMP-inhibited 3',5'-cyclic phosphodiesterase A | PDE3A |
|  |  | Alpha-1B adrenergic receptor | ADRA1B |
|  |  | Beta-2 adrenergic receptor | ADRB2 |
|  |  | Alpha-1D adrenergic receptor | ADRA1D |
|  |  | Mu-type opioid receptor | OPRM1 |
| MOL001460 | Cryptopin | Voltage-dependent L-type calcium channel subunit alpha-1S | CACNA1S |
|  |  | Nuclear receptor coactivator 1 | NCOA1 |
|  |  | cAMP and cAMP-inhibited cGMP 3',5'-cyclic phosphodiesterase 10A | PDE10A |
|  |  | 5-hydroxytryptamine 2A receptor | HTR2A |
|  |  | Sodium-dependent serotonin transporter | SLC6A4 |
| MOL001558 | Sesamin | Coagulation factor Xa | F10 |
|  |  | Prostaglandin G/H synthase 2 | PTGS2 |
|  |  | Sodium channel protein type 5 subunit alpha | SCN5A |
|  |  | G1/S-specific cyclin-D1 | CCND1 |
|  |  | Interleukin-10 | IL10 |
|  |  | Fatty acid synthase | FASN |
|  |  | Acetyl-CoA carboxylase 1 | ACACA |
|  |  | Glucose-6-phosphate 1-dehydrogenase | G6PD |
|  |  | Nitric oxide synthase, endothelial | NOS3 |
|  |  | Endothelin-converting enzyme 1 | ECE1 |
|  |  | Medium-chain specific acyl-CoA dehydrogenase, mitochondrial | ACADM |
|  |  | Cytochrome P450 2B6 | CYP2B6 |
|  |  | UDP-glucuronosyltransferase 1-1 | UGT1A1 |
|  |  | Sterol regulatory element-binding protein 1 | SREBF1 |
|  |  | NADPH oxidase 3 | NOX3 |
|  |  | NADPH oxidase 1 | NOX1 |
| Continue with the table below | | | |
| MOL001558 | Sesamin | Peroxisomal acyl-coenzyme A oxidase 1 | ACOX1 |
|  |  | ATP-citrate synthase | ACLY |
|  |  | Peroxisomal bifunctional enzyme | EHHADH |
|  |  | Methylglutaconyl-CoA hydratase, mitochondrial | AUH |
|  |  | Trifunctional enzyme subunit beta, mitochondrial | HADHB |
|  |  | 2,4-dienoyl-CoA reductase, mitochondrial | DECR1 |
|  |  | 3,2-trans-enoyl-CoA isomerase, mitochondrial | ECI1 |
| MOL001607 | ZINC03982454 | Progesterone receptor | PGR |
|  |  | Nuclear receptor coactivator 2 | NCOA2 |
|  |  | Sodium channel protein type 5 subunit alpha | SCN5A |
|  |  | Muscarinic acetylcholine receptor M3 | CHRM3 |
|  |  | Beta-2 adrenergic receptor | ADRB2 |
|  |  | Muscarinic acetylcholine receptor M1 | CHRM1 |
| MOL002211 | Eicosadienoic acid | Nuclear receptor coactivator 2 | NCOA2 |
| MOL002388 | Delphin_qt | Prostaglandin G/H synthase 1 | PTGS1 |
|  |  | Prostaglandin G/H synthase 2 | PTGS2 |
|  |  | Carbonic anhydrase II | CA2 |
|  |  | Phosphatidylinositol-4,5-bisphosphate 3-kinase catalytic subunit, gamma isoform | PIK3CG |
|  |  | Nuclear receptor coactivator 2 | NCOA2 |
| MOL002392 | Deltoin | Prostaglandin G/H synthase 1 | PTGS1 |
|  |  | Thrombin | F2 |
| MOL002392 | Deltoin | Sodium channel protein type 5 subunit alpha | SCN5A |
|  |  | Coagulation factor Xa | F10 |
|  |  | Prostaglandin G/H synthase 2 | PTGS2 |
|  |  | Acetylcholinesterase | ACHE |
|  |  | Alpha-1B adrenergic receptor | ADRA1B |
|  |  | Beta-2 adrenergic receptor | ADRB2 |
|  |  | Dipeptidyl peptidase IV | DPP4 |
|  |  | Trypsin-1 | PRSS1 |
| MOL002395 | Deoxyandrographolide | Prostaglandin G/H synthase 2 | PTGS2 |
|  |  | Progesterone receptor | PGR |
|  |  | Nuclear receptor coactivator 2 | NCOA2 |
|  |  | Nuclear receptor coactivator 1 | NCOA1 |
| MOL002398 | Karanjin | Prostaglandin G/H synthase 1 | PTGS1 |
|  |  | Estrogen receptor | ESR1 |
|  |  | Prostaglandin G/H synthase 2 | PTGS2 |
|  |  | Phosphatidylinositol-4,5-bisphosphate 3-kinase catalytic subunit, gamma isoform | PIK3CG |
|  |  | Serine/threonine-protein kinase Chk1 | CHEK1 |
| MOL002464 | 1-Monolinolein | Prostaglandin G/H synthase 1 | PTGS1 |
| MOL002501 | SCHEMBL119969 | Prostaglandin G/H synthase 2 | PTGS2 |
|  |  | Carbonic anhydrase II | CA2 |
|  |  | Nuclear receptor coactivator 2 | NCOA2 |
| MOL002514 | Sexangularetin | Nitric oxide synthase, inducible | NOS2 |
|  |  | Prostaglandin G/H synthase 1 | PTGS1 |
| MOL002514 | Sexangularetin | Androgen receptor | AR |
|  |  | Prostaglandin G/H synthase 2 | PTGS2 |
|  |  | Dipeptidyl peptidase IV | DPP4 |
|  |  | Cell division protein kinase 2 | CDK2 |
|  |  | Phosphatidylinositol-4,5-bisphosphate 3-kinase catalytic subunit, gamma isoform | PIK3CG |
|  |  | Trypsin-1 | PRSS1 |
| MOL002962 | 3-O-Methylviolanone | Nitric oxide synthase, inducible | NOS2 |
|  |  | Prostaglandin G/H synthase 1 | PTGS1 |
|  |  | Thrombin | F2 |
|  |  | Potassium voltage-gated channel subfamily H member 2 | KCNH2 |
|  |  | Sodium channel protein type 5 subunit alpha | SCN5A |
|  |  | Coagulation factor Xa | F10 |
|  |  | Prostaglandin G/H synthase 2 | PTGS2 |
|  |  | Nitric-oxide synthase, endothelial | NOS3 |
|  |  | Retinoic acid receptor RXR-alpha | RXRA |
|  |  | Acetylcholinesterase | ACHE |
|  |  | Alpha-1B adrenergic receptor | ADRA1B |
|  |  | Beta-2 adrenergic receptor | ADRB2 |
|  |  | Alpha-1D adrenergic receptor | ADRA1D |
|  |  | Estrogen receptor beta | ESR2 |
|  |  | Dipeptidyl peptidase IV | DPP4 |
|  |  | Cell division protein kinase 2 | CDK2 |
|  |  | Trypsin-1 | PRSS1 |
| MOL002962 | 3-O-Methylviolanone | Proto-oncogene serine/threonine-protein kinase Pim-1 | PIM1 |
|  |  | Cyclin-A2 | CCNA2 |
|  |  | Nuclear receptor coactivator 2 | NCOA2 |
|  |  | Calcium-activated potassium channel subunit alpha 1 | KCNMA1 |
| MOL003578 | Cycloartenol | Mineralocorticoid receptor | NR3C2 |
|  |  | Coagulation factor Xa | F10 |
|  |  | Prostaglandin G/H synthase 2 | PTGS2 |
|  |  | Coagulation factor VII | F7 |
|  |  | mRNA of Protein-tyrosine phosphatase, non-receptor type 1 | PTPN1 |
| MOL004135 | NSC643978 | Nitric oxide synthase, inducible | NOS2 |
|  |  | Prostaglandin G/H synthase 1 | PTGS1 |
|  |  | Thrombin | F2 |
|  |  | Androgen receptor | AR |
|  |  | Sodium channel protein type 5 subunit alpha | SCN5A |
|  |  | Peroxisome proliferator activated receptor gamma | PPARG |
|  |  | Prostaglandin G/H synthase 2 | PTGS2 |
|  |  | Nitric-oxide synthase, endothelial | NOS3 |
|  |  | Coagulation factor VII | F7 |
|  |  | Retinoic acid receptor RXR-alpha | RXRA |
|  |  | Alpha-1B adrenergic receptor | ADRA1B |
|  |  | Alpha-1D adrenergic receptor | ADRA1D |
|  |  | Estrogen receptor beta | ESR2 |
| MOL004135 | NSC643978 | Dipeptidyl peptidase IV | DPP4 |
|  |  | Mitogen-activated protein kinase 14 | MAPK1 |
|  |  | Glycogen synthase kinase-3 beta | GSK3B |
|  |  | Cell division protein kinase 2 | CDK2 |
|  |  | Serine/threonine-protein kinase Chk1 | CHEK1 |
| MOL004136 | Lycorine | Mu-type opioid receptor | OPRM1 |
|  |  | Neuronal acetylcholine receptor protein, alpha-7 chain | CHRNA7 |
| MOL004165 | CID11503965 | Coagulation factor Xa | F10 |
|  |  | Prostaglandin G/H synthase 2 | PTGS2 |
|  |  | mRNA of Protein-tyrosine phosphatase, non-receptor type 1 | PTPN1 |
| MOL005603 | Heptyl phthalate | Sodium-dependent noradrenaline transporter | SLC6A2 |
|  |  | Beta-2 adrenergic receptor | ADRB2 |
| MOL007991 | CHEMBL254186 | Nitric oxide synthase, inducible | NOS2 |
|  |  | Prostaglandin G/H synthase 1 | PTGS1 |
|  |  | Dopamine D1 receptor | DRD1 |
|  |  | Muscarinic acetylcholine receptor M3 | CHRM3 |
|  |  | Thrombin | F2 |
|  |  | Muscarinic acetylcholine receptor M1 | CHRM1 |
|  |  | Androgen receptor | AR |
|  |  | Muscarinic acetylcholine receptor M5 | CHRM5 |
|  |  | Prostaglandin G/H synthase 2 | PTGS2 |
|  |  | Retinoic acid receptor RXR-alpha | RXRA |
|  |  | 5-hydroxytryptamine 2A receptor | HTR2A |
| MOL007991 | CHEMBL254186 | Alpha-1A adrenergic receptor | ADRA1A |
|  |  | Sodium-dependent dopamine transporter | SLC6A3 |
|  |  | Beta-2 adrenergic receptor | ADRB2 |
|  |  | Alpha-1D adrenergic receptor | ADRA1D |
|  |  | Sodium-dependent serotonin transporter | SLC6A4 |
|  |  | Estrogen receptor beta | ESR2 |
|  |  | Dipeptidyl peptidase IV | DPP4 |
|  |  | Mitogen-activated protein kinase 14 | MAPK1 |
|  |  | Glycogen synthase kinase-3 beta | GSK3B |
|  |  | Cell division protein kinase 2 | CDK2 |
|  |  | Amine oxidase [flavin-containing] B | MAOB |
|  |  | Trypsin-1 | PRSS1 |
|  |  | Proto-oncogene serine/threonine-protein kinase Pim-1 | PIM1 |
|  |  | Cyclin-A2 | CCNA2 |
|  |  | Nuclear receptor coactivator 2 | NCOA2 |
| MOL009849 | ZINC05223929 | Prostaglandin G/H synthase 2 | PTGS2 |
|  |  | Coagulation factor Xa | F10 |
|  |  | Retinoic acid receptor RXR-alpha | RXRA |
| MOL012140 | Picrasidine D | Prostaglandin G/H synthase 1 | PTGS1 |
|  |  | Sodium channel protein type 5 subunit alpha | SCN5A |
|  |  | Prostaglandin G/H synthase 2 | PTGS2 |
|  |  | Nitric-oxide synthase, endothelial | NOS3 |
|  |  | Retinoic acid receptor RXR-alpha | RXRA |
| MOL012140 | Picrasidine D | CGMP-inhibited 3',5'-cyclic phosphodiesterase A | PDE3A |
|  |  | Gamma-aminobutyric acid receptor subunit alpha-1 | GABRA1 |
|  |  | Phosphatidylinositol-4,5-bisphosphate 3-kinase catalytic subunit, gamma isoform | PIK3CG |
| MOL012141 | Caribine | Muscarinic acetylcholine receptor M3 | CHRM3 |
|  |  | Muscarinic acetylcholine receptor M1 | CHRM1 |
|  |  | Sodium channel protein type 5 subunit alpha | SCN5A |
|  |  | Delta-type opioid receptor | OPRD1 |
|  |  | Alpha-1B adrenergic receptor | ADRA1B |
|  |  | Alpha-1D adrenergic receptor | ADRA1D |
|  |  | Mu-type opioid receptor | OPRM1 |
|  |  | Neuronal acetylcholine receptor protein, alpha-7 chain | CHRNA7 |
| MOL013219 | Strictosamide_qt | Prostaglandin G/H synthase 1 | PTGS1 |
|  |  | Thrombin | F2 |
|  |  | Potassium voltage-gated channel subfamily H member 2 | KCNH2 |
|  |  | Muscarinic acetylcholine receptor M1 | CHRM1 |
|  |  | Androgen receptor | AR |
|  |  | Sodium channel protein type 5 subunit alpha | SCN5A |
|  |  | Peroxisome proliferator activated receptor gamma | PPARG |
|  |  | Coagulation factor Xa | F10 |
|  |  | Prostaglandin G/H synthase 2 | PTGS2 |
| MOL013219 | Strictosamide_qt | Carbonic anhydrase II | CA2 |
|  |  | Coagulation factor VII | F7 |
|  |  | Retinoic acid receptor RXR-alpha | RXRA |
|  |  | Acetylcholinesterase | ACHE |
|  |  | Dipeptidyl peptidase IV | DPP4 |
|  |  | Trypsin-1 | PRSS1 |
|  |  | Nuclear receptor coactivator 1 | NCOA1 |

**Table S6.** Potential targets of BMK active compounds downloaded from Pharmmapper database

| Active compound | Target | Gene symbol | Norm Fit |
| --- | --- | --- | --- |
| Cholesterol | Vitamin D3 receptor | VDR | 1 |
| Cholesterol | Deoxyhypusine synthase | DHPS | 0.9998 |
| Cholesterol | Estrogen receptor | ESR1 | 0.9994 |
| Cholesterol | Protein S100-A8 | S100AB | 0.9993 |
| Cholesterol | Acyl-CoA-binding domain containing protein 7 | ACBD7 | 0.9993 |
| Cholesterol | Elongation factor 1-alpha | PGR | 0.9993 |
| Cholesterol | Lactotransferrin | LTF | 0.9993 |
| Cholesterol | Receptor-type tyrosine-protein phosphatase-like N | PTPRN | 0.9991 |
| Cholesterol | RNA-binding protein MEX3D | RKHD1 | 0.9988 |
| Cholesterol | Apoptosis regulator BAX | BAX | 0.9987 |
| Cholesterol | Ras-related C3 botulinum toxin substrate 1 | RAC1 | 0.9985 |
| Cholesterol | Interferon-induced, double-stranded RNA-activated protein kinase | EIF2AK2 | 0.9984 |
| Cholesterol | Calmodulin-like protein 3 | CALML3 | 0.9983 |
| Cholesterol | Cytochrome P450 3A4 | CYP3A4 | 0.9975 |
| Cholesterol | S-adenosylmethionine decarboxylase proenzyme | AMD1 | 0.9973 |
| Cholesterol | Cleavage and polyadenylation specificity factor subunit 4 | CPSF4 | 0.9966 |
| Cholesterol | E3 SUMO-protein ligase RanBP2 | RANBP2 | 0.9837 |
| Cholesterol | Dr1-associated corepressor | DRAP1 | 0.9486 |
| Cholesterol | Fibroblast growth factor 23 | FGF23 | 0.9373 |
| Cholesterol | Beta-elicitin cinnamomin | CHFR | 0.863 |
| Cholesterol | RuvB-like 1 | RUVBL1 | 0.8394 |
| Cholesterol | Nuclear factor Kappa-B | NFKB1 | 0.8049 |
| Cholesterol | Regulator of G-protein signaling 6 | RGS6 | 0.7789 |
| Cholesterol | Protein CBFA2T1 | MTG8 | 0.7551 |
| Cholesterol | Regulator of G-protein signaling 18 | RGS18 | 0.7518 |
| Cholesterol | ZINC FINGER PROTEIN SNAI1 | SNAI1 | 0.7509 |
| Cholesterol | Vitamin D-binding protein | GC | 0.75 |
| Cholesterol | Methionine synthase reductase, mitochondrial | MTRR | 0.7493 |
| Cholesterol | Death-associated protein kinase 3 | DAPK3 | 0.7492 |
| Cholesterol | Protein max, Transcription factor E2-alpha chimera | TCF3 | 0.7485 |
| Cholesterol | ADAM 17 | ADAM17 | 0.7485 |
| Cholesterol | 1,25-dihydroxyvitamin D(3) 24-hydroxylase, mitochondrial | CYP24A1 | 0.7475 |
| Cholesterol | Ribose-phosphate pyrophosphokinase 1 | PRPS1 | 0.747 |
| Katsutoxin | Transcription initiation factor TFIID subunit 9 | GLYR1 | 0.8257 |
| Katsutoxin | Putative oxidoreductase GLYR1 | HIBDL | 0.7689 |
| Katsutoxin | Coagulation factor XIII A chain | F13A1 | 0.7352 |
| Katsutoxin | Methylthioribose-1-phosphate isomerase | RBKS | 0.7269 |
| Stearin | RuvB-like 1 | RUVBL1 | 0.8352 |
| Stearin | Putative oxidoreductase GLYR1 | GLYR1 | 0.7689 |
| Stearin | Apolipoprotein C-II | APOC2 | 0.7493 |
| Stearin | Androgen receptor | AR | 0.7487 |
| Stearin | Rho GTPase-activating protein 11A | ARHGAP11A | 0.7337 |
| Stearin | Atrial natriuretic peptide receptor A | NPR1 | 0.7283 |
| Stearin | Kinesin-like protein KIF14 | KIF14 | 0.7031 |
| Chlorotoxin | Cleavage and polyadenylation specificity factor subunit 3 | CPSF3 | 0.7394 |
| Chlorotoxin | Dehydrogenase/reductase SDR family member 1 | DHRS1 | 0.7391 |
| Chlorotoxin | Acyl-CoA-binding domain-containing protein 6 | ACBD6 | 0.7385 |
| Chlorotoxin | Insulin-degrading enzyme | IDE | 0.737 |
| Chlorotoxin | Dihydrodipicolinate synthase | DAPA | 0.7362 |
| Chlorotoxin | Estrogen-related receptor gamma | NR3B3 | 0.7361 |
| Chlorotoxin | Vitamin D-binding protein | GC | 0.7344 |
| Chlorotoxin | tRNA pseudouridine synthase A | PUS1 | 0.7333 |
| Chlorotoxin | Delta(3,5)-Delta(2,4)-dienoyl-CoA isomerase, mitochondrial | ECH1 | 0.7329 |
| Chlorotoxin | Cullin-5 | CUL5 | 0.7317 |
| Bufotoxin | E3 SUMO-protein ligase RanBP2 | RANBP2 | 0.843 |
| Bufotoxin | Protection of telomeres protein 1 | POT1 | 0.7835 |
| Bufotoxin | Heterogeneous nuclear ribonucleoprotein R | HNRNPR | 0.7549 |
| Bufotoxin | Calpain-9 | CAPN9 | 0.7199 |
| Bufotoxin | Cold shock domain-containing protein E1 | CSDE1 | 0.7182 |
| 20-hexadecanoylingenol | Prostaglandin E2 receptor EP4 subtype | PTGER4 | 0.8785 |
| 20-hexadecanoylingenol | Short-chain specific acyl-CoA dehydrogenase, mitochondrial | ACADS | 0.8323 |
| 20-hexadecanoylingenol | Superoxide dismutase [Mn], mitochondrial | SOD2 | 0.8294 |
| 20-hexadecanoylingenol | Rho guanine nucleotide exchange factor 12 | ARHGEF12 | 0.7813 |
| 20-hexadecanoylingenol | Protection of telomeres protein 1 | POT1 | 0.7751 |
| 20-hexadecanoylingenol | Regulator of G-protein signaling 6 | RGS6 | 0.7556 |
| 20-hexadecanoylingenol | Acyl-CoA-binding domain containing protein 7 | ACBD7 | 0.7355 |
| 20-hexadecanoylingenol | Coagulation factor XIII A chain | F13A1 | 0.7352 |
| 20-hexadecanoylingenol | Protein kinase C alpha type | PRKCA | 0.729 |
| 20-hexadecanoylingenol | Interferon-induced guanylate-binding protein 1 | GBP1 | 0.7172 |

**Table S7.** The PDB information for core protein

| Target | PDBID | Ligand ID | POCASA | Docking pocket |
| --- | --- | --- | --- | --- |
| AVPR2 | 6UIN | 2CU | c, d | CYS176, TYR177, ILR178, ASN410, ALA414, TRP422 |
| BRD4 | 3MXF | JQ1 | a | PRO86, VAL87, LEU92, LEU94, ASN140 |
| CCND1 | 2W96 | GOL | inaccurate prediction | ARG87, LEU148, LYS149, ASN151 |
| CX3CL1 | 4XT3 | UNL | a | ChainA: LYS257, TYR177; ChainB: CYS34, SER33, PCA1, THR6 |
| MAPK1 | 5WP1 | B7S | a | ALA35, ASP106, MET108, CYS114, CME166 |
| MAPK8 | 3PZE | CFK | a, b | MET108, LEU110, MET111, ASP169 |
| p38MAPK | 1BL7 | SB4 | a | ILE147, HIS148, ASP168, ILE166 |
| PTGS2 | 4PH9 | HEM | inaccurate prediction | THR207, HIS208, THR213, ASN383, TYR386, HIS389 |
| TNF-α | 5MU8 | JNI | a | ASN19, GLU23, LEU26, TYR141,PHE144, VAL150 |
| TRPV1 | 5IS0 | 6ET | inaccurate prediction | TYR511, MET547, THR550 |

**
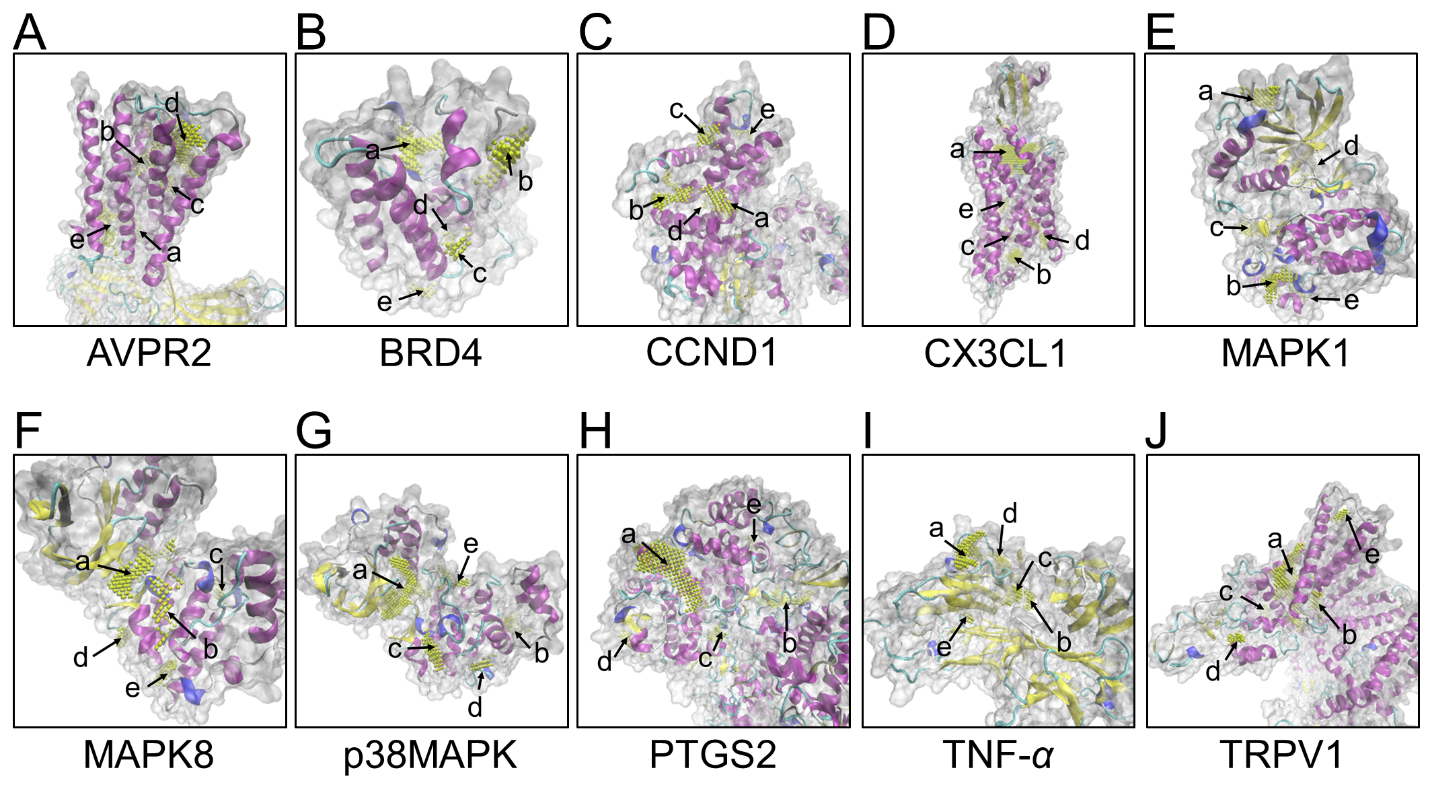
**

**Figure S1**. The docking pocket of target proteins predicted by POCASA v1.1, and the order of pocket volume is from large to small: a-e.
